# Use and evaluation of psychological interventions in specialist palliative care settings: results of a national online survey with psychologists and psycho-oncologists

**DOI:** 10.1186/s12904-026-02041-z

**Published:** 2026-03-06

**Authors:** Ricarda Scheiner, Isabel Sophie Burner-Fritsch, Martin Fegg, Berend Feddersen, Claudia Bausewein

**Affiliations:** 1 Department of Palliative Medicine, LMU University Hospital, Marchioninistr. 15, Munich, 81377 Germany; 2 Psychotherapy Clinic, Prof. Dr. Fegg and Colleagues, Sonnenstr. 10, Munich, 80331 Germany

**Keywords:** Psychological interventions, Palliative psychology, Psychologists, Psycho-oncologists, Palliative care

## Abstract

**Background:**

Psychologists play a key role as part of the multiprofessional palliative care team. Meaning-, dignity-centred and existential intervention approaches are designed to preserve dignity, strengthen the sense of meaning and alleviate existential distress at the end of life. However, little is known about psychological interventions used in caring for palliative patients and their relatives. This study aims to describe which psychological interventions are used in palliative care, and to what extent professional experience (years working as a psychologist/psycho-oncologist in a palliative care setting), palliative care setting, and further training in palliative care for psychologists influence the use of specific interventions of psychologists.

**Methods:**

German-wide online survey among psychologists/psycho-oncologists in adult specialist palliative care settings. Psychologists/psycho-oncologists who work in palliative care were recruited by e-mail through palliative care facilities or directly. Ordinal and logistic regressions were performed to examine the influence of professional experience, further training in palliative care for psychologists, and type of palliative care setting on the use of psychological interventions.

**Results:**

105/210 worked predominantly and 66/210 exclusively in palliative settings. 162/205 had further training in psycho-oncology and 36/205 in palliative care for psychologists. Dignity therapy (*M* = 1.93/elements thereof *M* = 3.24), meaning-centred therapy (*M* = 1.63/elements thereof *M* = 2.79), life review approaches (*M* = 2.08/elements thereof *M* = 3.14), or existential therapy (*M* = 1.37) were used less frequently than cross-school, low-threshold and non-specific interventions like resource collection (*M* = 6.54), exploration of previous coping strategies (*M* = 6.24), crisis intervention (*M* = 6.23) or psychoeducation (*M* = 5.67). Most common reason were unknown interventions or lack of resources.

Professional experience in the palliative setting increased the probability (OR = 1.147, *p* < .001) of using ‘(hypno)systemic-integrative’ interventions. Use of ‘meaning-dignity-existential’ interventions increased with professional experience (OR = 1.074, *p* = .045) and further training in palliative care for psychologists (OR = 2.341, *p* = .044).

**Conclusion:**

Despite ample evidence and recommendations, the majority of practitioners seem unaware of meaning, dignity and existential interventions or believe them to be unsuitable in palliative care. Targeted further training and a binding framework concept for palliative psychological care is needed.

**Supplementary Information:**

The online version contains supplementary material available at 10.1186/s12904-026-02041-z.

## What is already known about the topic

- Palliative patients and informal carers often suffer from distressing symptoms and burden that can be alleviated through palliative psychological support and targeted group-specific interventions.

## What this paper adds

- Psychologists and psycho-oncologists in Germany most frequently use non-specific cross-school, low-threshold, needs- and resource-oriented psychological interventions.
- Practitioners are less familiar with life review techniques, meaning- and dignity-centred interventions, logotherapy and existential analysis methods, and systemic and hypnotherapeutic approaches.
- The use of psychological interventions is influenced by further training in palliative care for psychologists and professional experience (years working as a psychologist/psycho-oncologist in a palliative care setting).

## Implications for practice, theory or policy

- To make palliative psychological care truly evidence based and comparable, a comprehensive conceptual framework with practice guidelines is required.
- The development of a consensus-based taxonomy of suitable psychological/psychotherapeutic approaches and interventions for palliative care could greatly benefit educational institutions, stakeholders, and future research projects.

## Introduction

Palliative patients suffer not only from physical symptoms but also from psychological, spiritual and social distress⁠ [1–4]. The psychological experiences of serious physical illness and approaching death can range from adequate emotional responses to psychiatric comorbidities and disorders. Psycho-existential distressing symptoms like anxiety, depression, hopelessness, demoralization, loss of meaning or dignity, discouragement, pointlessness, being trapped by the illness and even wishes to hasten death are common [1, 5–11]. Based on meta-analytical data, the combined prevalence of psychiatric syndromes (all types of depression, anxiety and adjustment disorders) in cancer patients and palliative care patients is reported to be 38.2% [12]. Consequently, the German S3 guideline for palliative medicine recommends that, in the event of a mental disorder, guideline-compliant diagnosis and therapy with psychotherapeutic, psychiatric and/or pharmacological expertise should also be offered at the end of life [13]. In a German cohort study existential distress (including demoralization, death anxiety and dignity-related distress) was described in 46.4% of patients with advanced cancer [1]. Informal carers are also affected in many ways by witnessing the illness, feeling overburdened or exhausted by provision of ongoing support, worries, fears or anticipatory grief [14–17]. To accurately and comprehensively identify and treat the wide range of symptoms and experienced distress in accordance with guidelines, the integration of palliative psychology expertise into multiprofessional palliative care teams (MPCT) is essential and highly recommended [13, 18–20]. Often, this seems to be optional, subject to widely varying national and international regulations, and is therefore repeatedly described as inadequate [21–28]. Furthermore, there seem to be significant shortcomings in the competence of non-psychological health care professionals in recognising psychological distress and in providing or arranging adequate psychological support [29–31]. Psychologists and psychotherapists themselves also express a need for additional training for example in systemic therapy, dignity therapy, narrative therapy, life story work, acceptance and commitment therapy, and mindfulness-based stress reduction [29]. Strada therefore suggests a very comprehensive skill set for palliative psychologists, which includes diagnostic, psychotherapeutic, psychiatric, pharmacological, palliative care, ethical-legal and cultural knowledge in dealing with the seriously ill, the dying and their relatives [32].

In Germany, conventional psychotherapy is regulated by law, is provided by licensed psychotherapists and aims to treat mental disorders and restore mental and physical functioning using guideline-based diagnostic and manualised interventions [33]. In contrast, psycho-oncological care addresses the complex, changing needs of cancer patients and is provided by physicians or academically trained psychosocial professionals (e.g., psychology or social pedagogy at master’s level qualifying for a scientifically recognised psychotherapeutic approach) who have completed certified additional psycho-oncological training [34]. Palliative psychology, as a relatively new subdiscipline of psychology, focuses on the treatment and support of seriously ill and dying patients with all forms of life-limiting illnesses, as well as the support of their relatives [35]. The steady development of this discipline in recent years was fostered by a basic curriculum [36], further training in palliative care for psychologists [37], the definition of a professional profile for psychologists in palliative care [35], and the opportunity to become certified as a specialist palliative care psychologist [38]. Scheiner et al. discuss the different qualification requirements and fields of activity of these psychological professions in greater detail [39].

The actual nature of psychologists’ work in palliative care, as well as its integration, has not been systematically researched to date [23, 26, 40]. Beyond classic psychological/psychotherapeutic intervention methods, specific interventions from the existential and humanistic approaches, such as life review procedures focusing on meaning and dignity, are described as being particularly effective and supportive in palliative care settings [41–50]. They unfold their positive effects for example by enhancing sense of dignity through narrative richness [51]. Quality of life and psychospiritual well-being can be improved by strengthening sense of meaning and experiencing inner peace [52, 53]. They may also enable life reassessment through remembrance and life review [46]. Recommendations and evidence for palliative psychological support and care can be summarised into seven key aspects: (a) early integration of palliative psychological expertise to reliably assess and record the psychological and existential symptom burden experienced by patients and their relatives to derive appropriate support needs (e.g. exploration, diagnostics and screening) [30, 32, 54]; (b) use of low-threshold, resource-oriented psychological interventions to support coping with illness and decision-making, and to promote self-efficacy (e.g. psychological counselling, ethical counselling, psychoeducation, resource collection, exploration and activation of coping strategies and impact techniques) [32, 55, 56]; (c) use of psychological interventions to support the management of distressing physical symptoms, such as pain, nausea, breathlessness or weakness (e.g. mindfulness-based stress reduction (MBSR) interventions [57, 58], hypnotherapy [59–61], body psychotherapy [62], interventions from embodiment research [63], relaxation techniques [64] and guided imagery [65]); (d) use of psychological and/or psychotherapeutic interventions which go beyond psychological counselling only and require specific (diagnostic) therapeutic skills to address distressing psychological symptoms, such as anxiety, depression, hopelessness, demoralisation, low mood, anger, shame, feeling trapped by the illness and loss of autonomy, identity or roles (e.g. cognitive behavioural therapy (CBT) [58, 66–68], acceptance and commitment therapy (ACT) [69, 70], systemic [71] and hypnotherapeutic interventions [72, 73], MBSR [57, 74–77] and psychodynamic interventions [78, 79]); (e) use of psychological interventions to address spiritual/existential distress (e.g. integration of spiritual aspects, meaning [53, 80, 81]- and dignity-centred interventions [82, 83], narrative approach [84], life review approaches [43, 85] and logotherapy/existential analysis interventions) [86, 87]; (f) use of psychological interventions for distressing social aspects, such as isolation or loneliness, worries about relatives or being a burden to others, financial worries and communication problems with relatives or team members (ethical and psychological counselling, promote successful communication with patients and relatives/in the multiprofessional team, systemic interventions) [32, 35, 71]; (g) use of interventions to prevent prolonged grief disorder (e.g. grief counselling for relatives, meaning- and dignity- centred interventions) [88–91]. Above all, meaning- and dignity-centred therapy, narrative therapy, life story work, existential therapy, systemic therapy and elements thereof can be considered a *specific palliative psychological approach*.

Therefore, the aim of this study is to describe which psychological interventions (PIs) are used by psychologists/psycho-oncologists in palliative care and to what extent professional experience (years working as a psychologist/psycho-oncologist in a palliative care setting), the palliative care setting, and further training in palliative care for psychologists influence the use of specific interventions.

## Methods

### Study design

A web-based online survey with psychologists/psycho-oncologists working in specialist palliative care settings for adults across Germany. The survey is reported following the Checklist for Reporting the Results of Internet e-Surveys (CHERRIES) [92]. The first part of these study results was recently published [39].

### Development and pre-testing of the questionnaire

Based on the literature and an online focus group discussion [93] with six experts in palliative psychology, a questionnaire was developed which allowed for different question formats (multiple choice with the option of multiple answers, open and closed questions, filter questions, rating questions and free text fields). Results of the content analysis with deductive category application of the focus group discussion allowed to select a manageable number of PIs that could be considered in the work with palliative patients and their relatives [94]. The entire survey contained 23 demographic questions, 45 questions on PIs, six questions on professional attitude and five questions on satisfaction facets. After a pre-test and a think-aloud protocol, the questionnaire was optimised [95].

### Recruitment process and description of the sample

Psychologists/psycho-oncologists who regularly work with adult patients and their relatives in German palliative care units within hospitals (PCU), palliative care support teams within hospitals (HST), specialist palliative home care (SPHC), inpatient hospices (IH) and psychological/psycho-oncological services or liaison services (POS) working in a consultative manner were invited to participate. All identifiable palliative care facilities and potential psychologists/psycho-oncologists working in specialist palliative care settings throughout Germany were contacted by email, informed about the study and invited to participate. A more detailed description of the recruitment process und resulting sample has been described in Scheiner et al. [39].

### Survey administration

A personalised access link to the questionnaire was sent to eligible participants via an invitation email from Lime Survey (software platform for web-based surveys) [96]. Psychologists/psycho-oncologists were informed about data protection at the beginning of the anonymised survey and had to confirm that their participation was voluntary before processing. It was possible to scroll back, take a break or to terminate the questionnaire at any time without giving reasons. The survey was active for six weeks and took place from end of October until mid-December 2023 with a reminder email after four weeks.

### Description of the variables reported in this article

1. Demographic characteristics: Gender, age, professional experience (PE) = years working as a psychologist/psycho-oncologist in a palliative care setting; degree at Master’s/Diploma level, training and further education, licence to (psychotherapy)practise; palliative setting, hours per week in palliative setting, integration into MPCT, job allocation/job sharing.
2. Self-assessment of the use of PIs: items PI01 to PI45 on a 7-point ordinal Likert scale from never (1) to very often (7), cut-off at 2 = rather rarely, then filter question with 7 response options on a nominal scale (1 = ‘I don’t know’, 2 = ‘not competent enough’, 3 = ‘patients/relatives rarely suitable for it’, 4 = ‘patient usually no longer able to’, 5 = ‘palliative care too short’, 6 = ‘not enough resources’, 7 = ‘other reasons’); if option 7 was selected, the individual reason could be specified in a free text field.

### Data analysis

The data were processed and analysed using IBM SPSS Statistics—Version 29.0.2.0 [20] [97]. After checking for missing values, influential data points, processing time, conspicuous time stamps and response patterns the raw data was adjusted according to predetermined exclusion criteria. Sample characteristics are reported in mean values, standard deviations, median or valid percentages depending on their scaling level. Response behaviour on the use of PIs (PI01 to PI45) is presented in absolute numbers, valid percentage values, mean values, standard deviations, standard errors of the mean values, median, skewness, kurtosis and range. Response behaviour for filter questions is presented in absolute numbers and the individual reasons from the free text fields are summarized as content-oriented analysis and integrated in the discussion. Intercorrelations between items of interest were calculated as Pearson for metric variables, Spearman (Rho) for ordinal variables and contingency measure Phi for dichotomous variables [98]. The following procedures were carried out to analyse how PE, predominantly/less working in a palliative setting, and the completion of further training in palliative care for psychologists (PCP) influence the use of specific interventions: a) creation of a dichotomous variable ‘predominantly palliative setting’ (PS) versus ‘less palliative setting’ (coding 0/reference category = less palliative setting, 1/control = PS); included in PS were those who indicated a predominant activity in PCU, SPHC, IH or HST; psychologists/psycho-oncologists who worked in POS, were only included if the POS hours were < than in PCU/HST/SPHC/IH or the hours were equally distributed (between POS and PCU/HST/SPHC/IH), as long as they were more than 10 h; outpatient settings without clear indicated working time were not included; b) theory-driven intuitive scale construction by combining those interventions that can most closely be associated with the (hypno)systemic-integrative approach (PI04, PI07, PI08, PI11, PI12, PI18, PI19, PI20, PI21, PI22, PI23) into ‘(hypno)systemic-integrative’ scale (ordinal 7-point Likert scale) for an ordinal regression using logit-link [99–102]; c) theory-driven intuitive scale construction by combining those interventions that can most closely be classified as meaning-, dignity-centred and existential approaches (PI33, PI34, PI35, PI36, PI37, PI38, PI39, PI40, PI45) into ‘meaning-dignity-existential’ scale [99]; due to a lack of normal and pronounced right-skewed distribution of these items even after combining them into one scale, the scale was dichotomised despite the risk of information loss [103] to facilitate interpretable binary logistic regression using enter method (coding 0/reference category = frequency low including never, almost never, rarely, 1/control = frequency high including very often, often, occasionally, rather rarely with an a priori defined cut-off at 3.5 out of 7, based on the conceptual midpoint separating low versus high frequency responses [104]) [102, 105, 106]; d) the variable PCP was coded 0/reference group = no further training in palliative care for psychologists, 1/control = further training in palliative care for psychologists. Imputation of the small proportion of missing values (1.86%) did not appear appropriate for this study design despite missing completely at random (χ^2^ (5040) = 4800.818, *p* = 0.992) [107–109]. Further exploratory analyses did not yield evidence of additional factors associated with the use of psychological interventions.

## Results

### Response rates and description of the sample

**Fig. 1:**
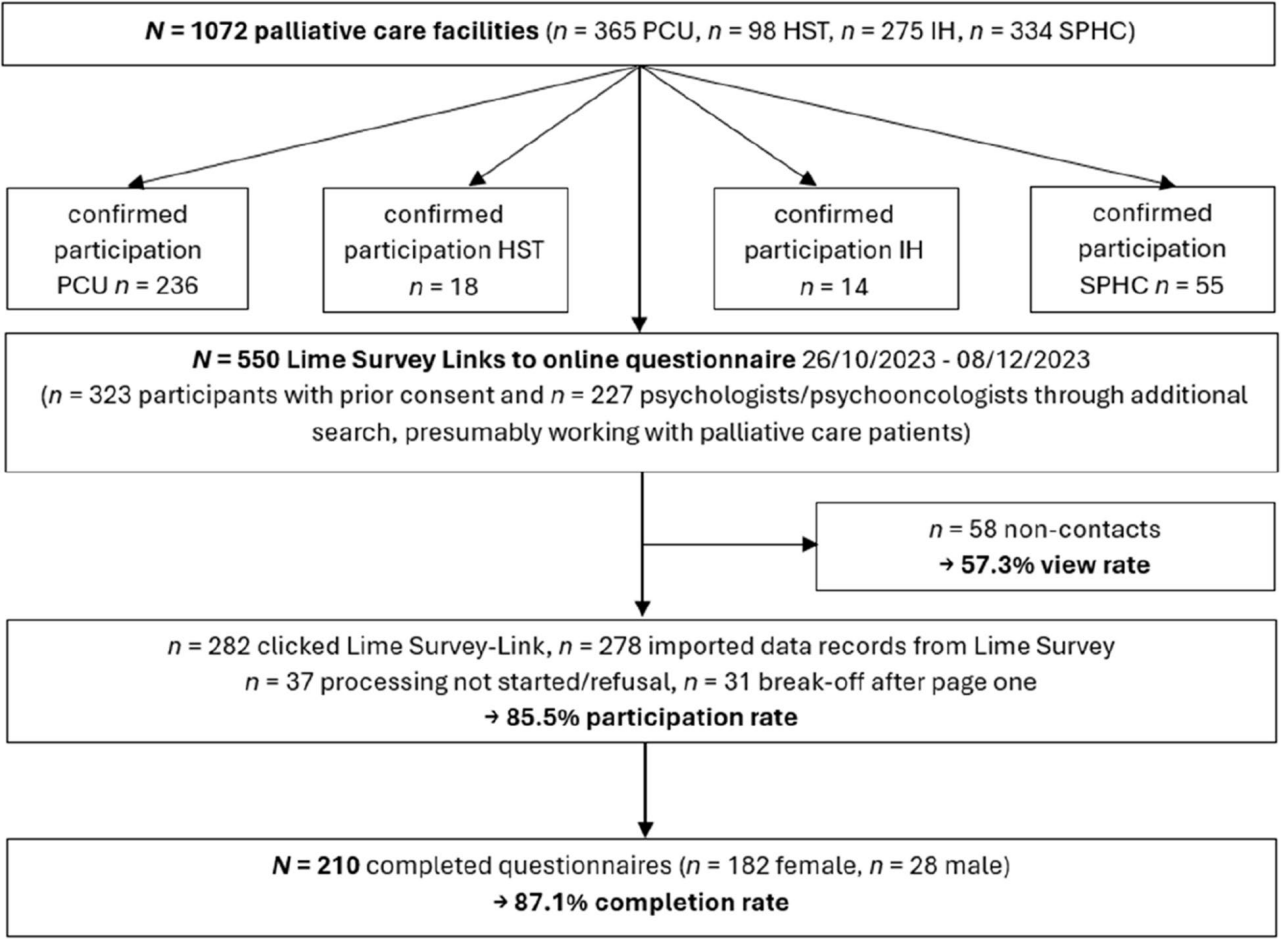
Flowchart of multi-stage sample recruitment and resulting sample reported according to CHERRIES Guidelines [92]. A total of *n* = 68 (24.5%) imported data sets were excluded due to missing data; PCU = Palliative Care Unit within a hospital, SPHC = specialist palliative home care, HST = palliative care support team within a hospital, IH = inpatient hospice, POS = psychological/psycho-oncological services or liaison services working in a consultative manner

Two hundred ten participants (86.7% female) with an average age of 44.8 years and PE of 6.15 years completed the questionnaire (view rate 57.3%, participation rate 85.5%, completion rate 87.1%), see Fig. 1 and Table 1. A small proportion (17.6%) had completed PCP, while 79% had undertaken further training in psycho-oncology. Half of the sample worked in a predominantly palliative setting and 57.1% were firmly integrated into a MPCT. Participants were most frequently employed both in a PCU and in the POS (36.7%), followed by employment in the POS (17.1%) or the PCU (15.2%) exclusively.

**Table 1:** Characteristics of the participating psychologists/psycho-oncologists (*N* = 210)

Descriptive Variable	*n*	*M (SD)*	*Median* (Min–Max)
Age^1^	209	44.77 (12.1)	43 (25–76)
Professional experience (as a psychologist/psycho-oncologist in palliative care setting) in years^1^	208	6.15 (4.9)	5 (0.5–25)
	***n* (valid %)**		**further details**
Gender (*n*^1^ = 210)			
female	182 (86.7%)		
male	28 (13.3%)		
Master’s level degree (*n*^1^ = 204)^2^			
Psychology	163 (79.9%)	*n* = 9/204 (4.4%) with two master’s degree *n* = 193/204 (94.6%) with one master’s degree	
Human medicine	20 (9.8%)		
Social pedagogy/social work	13 (6.4%)		
Other humanities or natural science degree programme	8 (3.9%)		
No master’s degree	2 (1%)		
License as a psychological psychotherapist (*n*^1^ = 202)^2^			
no licensed psychotherapist^3^	85 (42.1%)	*n* = 7 (3.5%) > one license as a psychological psychotherapist	
(Cognitive) behavioural therapy	79 (39.1%)		
Psychodynamic therapy/Depth psychology orientated	27 (13.4%)		
Systemic therapy	7 (3.5%)		
Medical psychotherapy	5 (2.5%)		
Humanistic oriented therapy	4 (2%)		
Psychotherapy for children and adolescents	3 (1.5%)		
Additional training and further education (*n*^1^ = 205)^2^			
Psycho-oncology	162 (79%)	Number of additional training and further education programmes *n* = 72 (35.1%) one *n* = 49 (23.9%) two *n* = 33 (16.1%) three *n* = 22 (10.7%) four *n* = 6 (2.9%) five *n* = 6 (2.9%) > five	
Palliative care for psychologists	36 (17.6%)		
Systemic counselling and therapy	33 (16.1%)		
Palliative care for psychosocial specialists	25 (12.2%)		
Hypnotherapy	22 (10.7%)		
Systemic counselling	17 (8.3%)		
Hypnosystemic therapy and counselling	6 (2.9%)		
Others	89 (43.4%)		
No training or further education	17 (8.3%)		
Palliative Setting (*n* = 210)			
PCU and POS	77 (36.7%)	*n* = 66 (31.4%) worked exclusively in a purely palliative setting (either only PCU, SPHC, HST or IH, or in a combination of these facilities) *n* = 105 (50%) respondents worked in a predominantly palliative setting	
POS	36 (17.1%)		
PCU	32 (15.2%)		
Other combination	17 (8.1%)		
SPH	13 (6.2%)		
PCU and HST and POS	8 (3.8%)		
IH	5 (2.4%)		
HST and POS	5 (2.4%)		
Other Setting (e.g. outpatient)	5 (2.4%)		
PCU and HST	4 (1.9%)		
PCU and SPHC and HST	3 (1.4%)		
PCU and SPHC	3 (1.4%)		
HST	2 (1%)		
Integration into the MPCT (*n* = 210)		psychologists in charge for palliative patients	
Firmly integrated	120 (57.1%)	*n* = 83 (39.5%) solely	
Consultative	56 (26.7%)	*n* = 50 (23.8%) two	
Firmly and consultative	23 (11%)	*n* = 38 (18.1%) three	
Other form of cooperation	11 (5.2%)	*n* = 41 (19.5%) more than three and depending on Setting	

^1^*N’s* range from 202 to 210 due to randomly missing data

^2^*N* varies due to the possibility of multiple answers. *PCU* Palliative Care Unit within a hospital, *SPHC* specialist palliative home care, *HST* palliative care support team within a hospital, *IH* inpatient hospice, *POS* psychological/psycho-oncological services or liaison services working in a consultative manner, *MPCT* multiprofessional palliative care team

^3^*n* = 18 (8.9%) indicated psychotherapy according to the ‘Heilpraktikergesetz’, which is not part of the legal psychotherapeutic licensing training programmes

### Psychological interventions

Exact wording and response behaviour of the participants on the use of PIs (PI01 to PI45) can be seen in Table 2. Table 3 provides a more detailed analysis of the items. Low-threshold and rather non-specific interventions like resource collection, psychological counseling, crisis intervention or psychoeducation showed a strong tendency towards agreement (left-skewed) and little discriminatory power, as not all seven response categories were used. Interventions from the fields of systemic therapy and counselling, MBSR, specific psycho-oncological interventions, ethical counselling or body psychotherapy methods were in the middle range. Interventions of the meaning-, dignity- and existential approach were particularly right skewed and discriminated between the respondents to a limited extent. Large (*r* = 0.37 to *r* = 0.45, *p* < 0.001, e.g. PI33 and PI36, PI34 and PI36, PI34 and PI37, PI35 and PI37, PI35 and PI38, PI35 and PI40, PI37 and PI39, PI38 and PI40) to strong (*r* ≥ 0.50, *p* < 0.001, e.g. PI38 and PI39, PI33 and PI34, PI33 and PI34, PI35 and PI36, PI36 and PI37, PI36 and PI38) effect sizes of the intercorrelations (see Appendix A—Table) between some of the examined items revealed initial conclusions about strength and direction of relationships and allowed items to be selected for scale construction. Table 4 provides an overview of categories of similarly frequently used interventions.

**Table 2:** Response behaviour to the items psychological intervention PI01 to PI45 among psychologists/psycho-oncologists (*N* = 210); each question began with ‘How often do you use’ and ended with ‘in your work with the seriously ill, the dying and/or their relatives/carers?’

**Item psychological intervention PI01 to PI 45**	***n***^a^	proportion of agreement *n* (valid %)						
		**never (1)**	**almost never (2)**	**rarely (3)**	**rather rarely (4)**	**occasionally (5)**	**often (6)**	**very often (7)**
PI01 ‘guideline-based psychological diagnostics/screening’ (e.g. with Distress-Thermometer, HADS, GHQ, PO-Bado, SMILE, EORTC QLQ-C30, S3 Guideline Palliative Medicine Depression/Anxiety, etc.)	210	38 (18.1%)	31 (14.8%)	18 (8.6%)	11 (5.2%)	42 (20%)	25 (11.9%)	45 (21.4%)
PI02 ‘explorative diagnostics without guidelines’ (e.g. in the form of information gathering during discussions, exchanges within the multiprofessional team, researching previous documentation or observations)	210	3 (1.4%)	3 (1.4%)	-	1 (0.5%)	10 (4.8%)	39 (18.6%)	154 (73.3%)
PI03 ‘request for an order’ (making contact without an order and obtaining an order from the patient or relatives in the initial contact)	210	16 (7.6%)	8 (3.8%)	6 (2.9%)	9 (4.3%)	33 (15.7%)	55 (26.2%)	83 (39.5%)
PI04 ‘order clarification’ (clarification or collection of current concerns/issues/goals)	210	-	-	-	3 (1.4%)	24 (11.4%)	91 (43.3%)	92 (43.8%)
PI05 ‘situation analysis/behavioural analysis’ (in the sense of a conversation-based exploration of current distress and associated feeling/thinking/behavioural patterns)	210	10 (4.8%)	19 (9%)	6 (2.9%)	20 (9.5%)	42 (20%)	58 (27.6%)	55 (26.2%)
PI06 ‘exploration of previous coping strategies’ (analysis and evaluation of previous coping strategies, what has helped so far, what has helped less)	210	-	2 (1%)	1 (0.5%)	2 (1%)	27 (12.9%)	85 (40.5%)	93 (44.3%)
PI07 ‘resource collection’ (in the sense of an exploration of existing and future resources, resource activation)	210	-	-	1 (0.5%)	1 (0.5%)	15 (7.1%)	59 (28.1%)	134 (63.8%)
PI08 ‘biography work’ (e.g. genogram, biographical-narrative interventions, etc.)	210	4 (1.9%)	8 (3.8%)	7 (3.3%)	15 (7.1%)	56 (26.7%)	78 (37.1%)	42 (20%)
PI09 ‘psychoanalytical exploration/processing’ (in the sense of uncovering and/or interpreting unconscious/partially conscious mental processes, e.g. raising awareness of defence processes, transference/countertransference or working with dreams)	210	57 (27.1%)	43 (20.5%)	26 (12.4%)	20 (9.5%)	50 (23.8%)	10 (4.8%)	4 (1.9%)
PI10 ‘psychological counselling’ (e.g. counselling on palliative care, dealing with children and adolescents of seriously ill relatives; recommending/referring support services)	210	-	-	-	1 (0.5%)	26 (12.4%)	89 (42.4%)	94 (44.8%)
PI11 ‘interventions to promote successful communication with patients and relatives’ (e.g. conducting and/or moderating couple discussions, family discussions; taking on a mediating role between patients and relatives)	210	1 (0.5%)	5 (2.4%)	3 (1.4%)	10 (4.8%)	94 (44.8%)	70 (33.3%)	27 (12.9%)
PI12 ‘interventions to promote successful communication in the multiprofessional team’ (e.g. conducting and/or moderating case discussions; taking on a mediating role in the multi-professional team; taking on a mediating role between the team and patients/relatives, e.g. sensitising them to ‘double awareness’)	209	11 (5.3%)	14 (6.7%)	16 (7.7%	14 (6.7%)	84 (40.2%)	53 (25.4%)	17 (8.1%)
PI13 ‘ethical counselling’ (e.g. conducting and/or moderating ethical case discussions; taking an ethical counselling position within the multiprofessional team or for patients and relatives)	207	45 (21.7%)	26 (12.6%)	26 (12.6%)	25 (12.1%)	68 (32.9%)	14 (6.8%)	3 (1.4%)
PI14 ‘psychoeducation’ (in the sense of didactic-psychotherapeutic interventions to convey information)	208	4 (1.9%)	-	5 (2.4%)	12 (5.8%)	56 (26.9%)	84 (40.4%)	47 (22.6%)
PI15 ‘crisis intervention**’**(broadly defined, in the sense of emotional relief, reflection on the cause of the crisis, reintegration, e.g. by means of containing, empathic listening, normalisation, validation, etc.)	209	-	-	3 (1.4%)	-	36 (17.2%)	77 (36.8%)	93 (44.5%)
PI16 ‘client-centred conversation’ (in the sense of Carl Rogers and the conditions he named: acceptance = unconditional positive regard, empathy = empathetic understanding/sensitive, active listening, congruence = authenticity/genuineness/honesty; also includes the general effective factors according to Klaus Grawe, such as the therapeutic relationship, resource activation, problem actualisation, motivational clarification and problem solving)	208	3 (1.4%)	2 (1%)	1 (0.5%)	5 (2.4%)	13 (6.3%)	54 (26%)	130 (62.5%)
PI17 ‘elements from humanistic psychotherapy’ (e.g. experiencing, focusing, self-actualisation)	206	37 (18%)	14 (6.8%)	22 (10.7%)	18 (8.7%)	60 (29.1%)	36 (17.5%)	19 (9.2%)
PI18 ‘systemic questions’ (in the sense of systemic dialogue, e.g. with scaling questions, percentage questions, circular questions to stimulate a change of perspective, miracle questions, aggravation questions, etc.)	210	9 (4.3%)	8 (3.8%)	15 (7.1%)	14 (6.7%)	78 (37.1%)	57 (27.1%)	29 (13.8%)
PI19 ‘impact techniques’ (in the sense of psychotherapeutic use of images, metaphors, symbols, myths, music, poetry, etc.; amplification; visualisation; reframing, etc.)	209	8 (3.8%)	11 (5.3%)	4 (1.9%)	20 (9.6%)	75 (35.9%)	56 (26.8%)	35 (16.7%)
PI20 ‘interventions from systemic therapy and counselling’ (e.g. family board, timeline = working with a timeline, sculpture work, life flow model, lifeline, mini-max interventions, etc.)	210	42 (20%)	31 (14.8%)	25 (11.9%)	37 (17.6%)	51 (24.3%)	18 (8.6%)	6 (2.9%)
PI21 ‘systemic palliative psychotherapy’ according to Sandra Burgstaller (psychotherapeutic support based on the palliative transition model)	203	158 (77.8%)	12 (5.9%)	1 (0.5%)	9 (4.4%)	18 (8.9%)	4 (2%)	1 (0.5%)
PI22 ‘hypnosystemic interventions’ according to Gunther Schmidt (e.g. work with inner parts, also in the sense of ego-state therapy; hypnosystemic communication for targeted focussing of attention, etc.)	206	89 (43.2%)	22 (10.7%)	8 (3.9%)	17 (8.3%)	50 (24.3%)	11 (5.3%)	9 (4.4%)
PI23 ‘hypnotherapeutic interventions’ according to Milton Erickson (e.g. hypnotherapeutic communication with pacing & leading, hypnotic trance, (post-)hypnotic suggestions, guidance on self-hypnosis, etc.)	208	97 (46.6%)	15 (7.2%)	12 (5.8%)	11 (5.3%)	54 (26%)	10 (4.8%)	9 (4.3%)
PI24 ‘cognitive elements from behavioural therapy’ (e.g. cognitive restructuring; confrontation in sensu (e.g. thinking to the end in the case of anxiety); Socratic dialogue, control dispute, etc.)	209	9 (4.3%)	9 (4.3%)	9 (4.3%)	19 (9.1%)	74 (35.4%)	54 (25.8%)	35 (16.7%)
PI25 ‘behavioural elements from behavioural therapy’ (e.g. behavioural activation, such as developing behavioural coping strategies; behavioural inhibition, such as stopping rumination; role-playing; problem-solving training, skills training, etc.)	209	18 (8.6%)	17 (8.1%)	6 (2.9%)	21 (10%)	77 (36.8%)	51 (24.4%)	19 (9.1%)
PI26 ‘interventions from acceptance and commitment therapy (ACT)’ (e.g. value orientation; promotion of acceptance and willingness; cognitive de-fusion; mindfulness, etc.)	207	13 (6.3%)	7 (3.4%)	6 (2.9%)	7 (3.4%)	74 (35.7%)	74 (35.7%)	26 (12.6%)
PI27 ‘psycho-oncological interventions according to Diegelmann and Isermann’ (e.g. TRUST = techniques for resource-focussed and symbolic trauma processing, PEACE = Posttraumatic Growth, Energy, Awareness, Confidence, Equilibrium, CIPBS® = Conflict Imagination Painting and Bilateral Stimulation; luminous flow exercise, ZEN circle, etc.)	207	87 (42%)	17 (8.2%)	10 (4.8%)	15 (7.2%)	47 (22.7%)	14 (6.8%)	17 (8.2%)
PI28 ‘trauma therapeutic interventions’ (e.g. narrative exposure therapy; elements from EMDR = Eye Movement Desensitisation and Reprocessing; IRRT = Imagery Rescripting and Reprocessing, etc.)	209	114 (54.5%)	35 (16.7%)	6 (2.9%)	17 (8.1%)	25 (12%)	8 (3.8%)	4 (1.9%)
PI29 ‘body psychotherapeutic interventions’ (e.g. imaginative body therapy; breathing therapy; work according to Ulfried Geuter with the elements of perceiving and sensing, awareness and presence, exploring and discovering, activating and expressing, regulating and modulating, centring and grounding, touching and holding, staging and interacting, embodying and acting, reorganising and transforming)	210	67 (31.9%)	25 (11.9%)	11 (5.2%)	13 (6.2%)	61 (29%)	21 (10%)	12 (5.7%)
PI30 ‘interventions from embodiment research’ (e.g. work with elements from the ZRM® = Zurich Resource Model according to Maja Storch)	204	137 (67.2%)	13 (6.4%)	5 (2.5%)	14 (6.9%)	25 (12.3%)	6 (2.9%)	4 (2%)
PI31 ‘interventions from MBSR (Mindfulness Based Stress Reduction according to Jon Kabat-Zinn)’ (e.g. mindfulness exercises, meditation, imagination, body scan, yoga/mindfulness yoga)	207	18 (8.2%)	17 (8.2%)	8 (3.9%)	9 (4.3%)	85 (41.1%)	48 (23.2%)	22 (10.6%)
PI32 ‘relaxation techniques’ (e.g. PMR = progressive muscle relaxation, autogenic training, biofeedback, fantasy journeys)	209	9 (4.3%)	10 (4.8%)	10 (4.8%)	15 (7.2%)	78 (37.3%)	63 (30.1%)	24 (11.5%)
PI33 ‘manualised dignity-centred therapy = Dignity Therapy according to Harvey Chochinov’ (processing all questions, audio recording the conversations, transcribing the conversations, editing the transcript, reading aloud and correcting the transcript if necessary, handing over the text document to the patient or relatives)?	206	137 (66.5%)	22 (10.7%)	10 (4.9%)	8 (3.9%)	22 (10.7%)	5 (2.4%)	2 (1%)
PI34 ‘elements from Harvey Chochinov’s dignity-centred therapy’ (e.g. Dignity Talk; use of individual questions from dignity-centred therapy; creating a legacy in the form of letters/audio/video recordings, etc.)	206	76 (36.9%)	21 (10.2%)	13 (6.3%)	11 (5.3%)	51 (24.8%)	27 (13.1%)	7 (3.4%)
PI35 ‘manualised meaning-centred interventions according to William Breitbart’ (e.g. meaning-centred psychotherapy = meaning-centred individual and group psychotherapy or SPT-PC = meaning-centred psychotherapy palliative care)	205	162 (79%)	6 (2.9%)	8 (3.9%)	10 (4.9%)	18 (8.8%)	-	1 (0.5%)
PI36 ‘manualised meaning-centred interventions’ (e.g. CALM = Managing Cancer and Living Meaningfully; SMiLE = Schedule for Meaning in Life Evaluation; Outlook = Intervention to prepare for the end of life; meaning-centred interventions from logotherapy and existential analysis)	208	129 (62%)	17 (8.2%)	8 (3.8%)	7 (3.4%)	34 (16.3%)	10 (4.8%)	3 (1.4%)
PI37 ‘elements of meaning-centred interventions’ (use of individual topics/questions from the interventions Meaning Centred Psychotherapy = meaning-centred individual and group psychotherapy or SPT-PC = meaning-centred psychotherapy palliative care, CALM = Managing Cancer and Living Meaningfully, SMiLE = Schedule for Meaning in Life Evaluation, Outlook = intervention to prepare for the end of life, meaning-centred interventions of logotherapy and existential analysis)	204	100 (49%)	17 (8.3%)	9 (4.4%)	7 (3.4%)	51 (25%)	14 (6.9%)	6 (2.9%)
PI38 ‘manualised life review approaches**’** (e.g. Short-Term Life Review; Life Review Therapy)	207	142 (68.6%)	10 (4.8%)	6 (2.9%)	5 (2.4%)	34 (16.4%)	9 (4.3%)	1 (0.5%)
PI39 ‘elements from life review approaches’ (use of individual questions/topics from the Short-Term Life Review or Life Review Therapy)	205	98 (47.8%)	4 (2%)	6 (2.9%)	3 (1.5%)	61 (29.8%)	29 (14.1%)	4 (2%)
PI40 ‘manualised existential psychotherapy**’** (e.g. EBT = Existential Behavioural Therapy as a group programme, etc.)	202	173 (85.6%)	7 (3.5%)	8 (4%)	8 (4%)	3 (1.5%)	2 (1%)	1 (0.5%)
PI41 ‘grief counselling for relatives’ (in the sense of supportive counselling with relatives of the deceased to prevent aggravated or prolonged grief; (hypnosystemic) grief counselling; recommendation/referral to grief counselling services)	208	13 (6.3%)	15 (7.2%)	8 (3.8%)	13 (6.3%)	69 (33.2%)	54 (26%)	36 (17.3%)
PI42 ‘catathymic-imaginative psychotherapy’ (guided affective imagery = depth psychology-based work with daydream images)	206	169 (82%)	11 (5.3%)	4 (1.9%)	8 (3.9%)	13 (6.3%)	1 (0.5%)	-
PI43 ‘other/creative methods/artistic therapies’ (e.g. art therapy, music therapy, singing, painting, dancing, movement, poetry, etc.)	207	101 (48.8%)	35 (16.9%)	15 (7.2%)	11 (5.3%)	30 (14.5%)	11 (5.3%)	4 (1.9%)
PI44 … (or another appropriately trained person from within or outside the organisation) use ‘animal-assisted therapy’ (e.g. with dogs, horses or other animals)	206	147 (71.4%)	3 (1.5%)	2 (1%)	7 (3.4%)	31 (15%)	12 (5.8%)	4 (1.9%)
PI45 ‘integration of spiritual aspects into therapeutic activities’ (e.g. addressing questions of faith or spiritual needs)	209	12 (5.7%)	5 (2.4%)	6 (2.9%)	9 (4.3%)	82 (39.2%)	65 (31.1%)	30 (14.4%)

^a^The varying *n*’s are due to randomly missing values. Assessment of the items on 7-point Likert scale from ‘never’ to ‘very often’. If the answer category ‘rather rarely’ to ‘never’ was selected, the reason was asked in a next step (adaptive testing)

**Table 3:** Mean values, standard deviations, standard errors of the mean values, median, skewness, kurtosis and range of the analysed items (*N* = 210)

**Item**	***n***^a^	***M***	***SD***	***SE***	***Median***	**25th–75th Percentile**	**Skewness**	**Kurtosis**	**Range**
PI01 ‘guideline-based psychological diagnostics/screening’	210	4.16	2.22	.15	5	2–6	-.142	−1.464	6
PI02 ‘explorative diagnostics without guidelines’	210	6.55	1.05	.07	7	6–7	−3.561	14.471	6
PI03 ‘request for an order’	210	5.53	1.82	.13	6	5–7	−1.383	.902	6
PI04 ‘order clarification’	210	6.30	.72	.05	6	6–7	-.744	.070	3
PI05 ‘situation analysis/behavioural analysis’	210	5.19	1.76	.12	6	4–7	-.964	-.039	6
PI06 ‘exploration of previous coping strategies’	210	6.24	.87	.06	6	6–7	−1.654	4.889	5
PI07 ‘resource collection’	210	6.54	.69	.05	7	6–7	−1.641	3.294	4
PI08 ‘biography work’	210	5.44	1.36	.09	6	5–7	−1.276	1.707	6
PI09 ‘psychoanalytical exploration/processing’	210	3.04	1.76	.12	3	1–5	.325	−1.211	6
PI10 ‘psychological counselling’	210	6.31	.70	.05	6	6–7	-.610	-.463	3
PI11 ‘promote successful communication with patients and relatives’	210	5.42	1.03	.07	5	5–6	−1.080	2.962	6
PI12 ‘promote successful communication in the multiprofessional team’	209	4.78	1.54	.11	5	4–6	-.957	0.319	6
PI13 ‘ethical counselling’	207	3.48	1.75	.12	4	2–5	-.159	−1.323	6
PI14 ‘psychoeducation’	208	5.67	1.15	.08	6	5–6	−1.489	3.822	6
PI15 ‘crisis intervention’	209	6.23	0.84	.06	6	6–7	−1.055	1.455	4
PI16 ‘client-centred conversation’	208	6.39	1.09	.08	7	6–7	−2.815	9.609	6
PI17 ‘elements from humanistic psychotherapy’	206	4.14	1.95	.14	5	2.75–6	-.391	−1.094	6
PI18 ‘systemic questions’	210	5.05	1.49	.10	5	5–6	−1.042	.847	6
PI19 ‘impact techniques’	209	5.16	1.48	.10	5	5–6	−1.116	1.155	6
PI20 ‘interventions from systemic therapy and counselling’	210	3.49	1.76	.12	4	2–5	-.019	−1.171	6
PI21 ‘systemic palliative psychotherapy’	203	1.68	1.45	.10	1	1–1	1.946	2.298	6
PI22 ‘hypnosystemic interventions’	206	2.93	2.02	.14	2	1–5	.434	−1.348	6
PI23 ‘hypnotherapeutic interventions’	208	2.88	2.04	.14	2	1–5	.458	−1.362	6
PI24 ‘cognitive elements from behavioural therapy’	209	5.11	1.51	.10	5	5–6	−1.051	.920	6
PI25 ‘behavioural elements from behavioural therapy’	209	4.68	1.69	.12	5	4–6	-.912	-.025	6
PI26 ‘interventions from acceptance and commitment therapy (ACT)’	207	5.16	1.52	.11	5	5–6	−1.443	1.749	6
PI27 ‘psycho-oncological interventions according to Diegelmann and Isermann’	207	3.14	2.16	.15	2	1–5	.366	−1.407	6
PI28 ‘trauma therapeutic interventions’	209	2.25	1.74	.12	1	1–4	1.143	-.070	6
PI29 ‘body psychotherapeutic interventions’	210	3.41	2.07	.14	4	1–5	.081	−1.517	6
PI30 ‘interventions from embodiment research’	204	2.07	1.75	.12	1	1–3	1.314	.238	6
PI31 ‘interventions from MBSR’	207	4.73	1.71	.12	5	4–6	-.946	-.004	6
PI32 ‘relaxation techniques’	209	5.05	1.47	.10	5	5–6	−1.154	1.097	6
PI33 ‘manualised dignity-centred therapy’	206	1.93	1.57	.11	1	1–2	1.539	1.016	6
PI34 ‘elements from dignity-centred therapy’	206	3.24	2.08	.15	3	1–5	.184	−1.577	6
PI35 ‘manualised meaning-centred interventions according to William Breitbart’	205	1.63	1.35	.09	1	1–1	1.934	2.350	6
PI36 ‘manualised meaning-centred interventions (CALM, SMiLE, Outlook, etc.)’	208	2.24	1.83	.13	1	1–4	1.084	-.440	6
PI37 ‘elements of meaning-centred interventions’	204	2.79	2.04	.14	2	1–5	.520	−1.384	6
PI38 ‘manualised life review approaches’	207	2.08	1.77	.12	1	1–3	1.217	-.243	6
PI39 ‘elements from life review approaches’	205	3.14	2.17	.15	3	1–5	.167	−1.773	6
PI40 ‘manualised existential psychotherapy’	202	1.37	1.04	.07	1	1–1	3.100	9.623	6
PI41 ‘grief counselling for relatives’	208	5.00	1.68	.12	5	5–6	-.995	.254	6
PI42 ‘catathymic-imaginative psychotherapy’	206	1.49	1.18	.08	1	1–1	2.362	4.180	5
PI43 ‘other/creative methods/artistic therapies’	207	2.43	1,80	.13	2	1–4	.950	-.512	6
PI44 ‘animal-assisted therapy’	206	2.15	1.91	.13	1	1–4	1.205	-.271	6
PI45 ‘integration of spiritual aspects into therapeutic activities’	209	5.20	1.47	.10	5	5–6	−1.417	2.041	6

^a^The varying *n*’s are due to randomly missing values. Assessment of the items on 7-point Likert scale from ‘never’ to ‘very often’ for the items psychological intervention

**Table 4:**
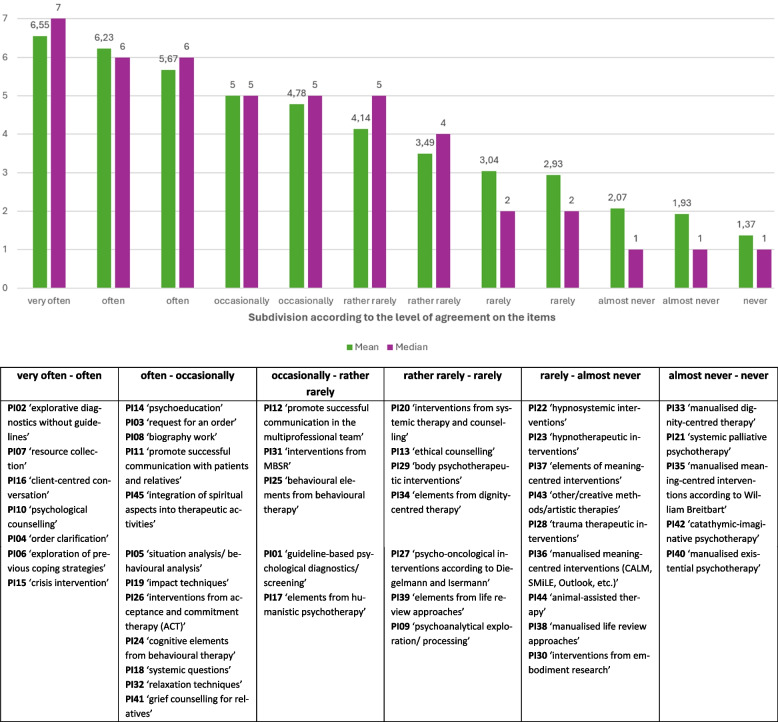
Items PI01 to PI45 grouped in descending order according to the level of the mean values and median within the categories (tendency to agree), *N* = 210

Assessment of the items on 7-point Likert scale from ‘never’ (1) to ‘very often’ (7). For this grouping, the highest and lowest mean values/medians from the neighbouring evaluation categories were visualised as examples; within the categories shown, the items below are arranged in descending order. The categories were visualised using the highest and lowest mean values between two adjacent response categories on the Likert scales. The first intervention in the list below has the highest mean and median; the last intervention has the lowest. The mean and median values of the interventions in between lie between these two values

### Influence of professional experience, palliative care setting and further training in palliative care for psychologists on the use of (hypno)systemic-integrative interventions

**Fig. 2:**
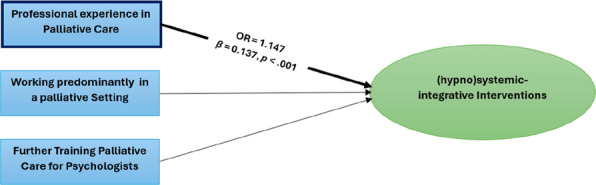
*Ordinal* regression model for predicting the utilisation of (hypno)systemic-integrative interventions. The ordinal regression model (logit link) was statistically significant, χ^2^(3) = 30.481, *p* <.001, with high goodness of fit (Pearson, χ^2^(3021) = 2693.376, *p* >.05; Deviation measure, χ^2^(3021) = 895.147, *p* >.05), but with low variance explained by Nagelkerke’s *R*.^*2*^ =.140. Only professional experience in the palliative setting had a positive influence on the use of (hypno)systemic-integrative interventions, with a *β* = 0.137, *p* <.001 (95% CI [.084,.190]), *N* = *203*

As an intermediate step a theory-driven intuitive scale was calculated (see Appendix B—Table) from the mean values of the items PI04, PI07, PI08, PI11, PI12, PI18, PI19, PI20, PI21, PI22 and PI23 (*M* = 4.53, *MIC* = 0.220, *Cronbach’s α* = 0.761). The data for the ordinal regression analysis met the assumptions of independent observations, no perfect multicollinearity and parallelism [−2 Log-Likelihood = 980.342, χ2(141) = 119.114, *p* = 909]. The statistically significant model [χ2(3) = 30.481, *p* < 0.001], with high goodness of fit [Pearson χ2(3021) = 2693.376, *p* > 0.05; deviation measure χ2(3021) = 895.147, *p* > 0.05] explained low proportion of variance (Nagelkerke’s *R*^*2*^ = 0.140) (see Fig. 2 and Appendix C—Table). Higher PE increased the probability (OR = 1.147, CI [1.088, 1.209]) of being classified in a higher rating category of the utilisation of (hypno)systemic-integrative interventions, with a *β* = 0.137 (*p* < 0.001, 95% CI [0.084, 0.190]). PCP and PS showed no significant influence.

### Influence of professional experience, palliative care setting and further training in palliative care for psychologists on the use of meaning-dignity-existential interventions

**Fig. 3:**
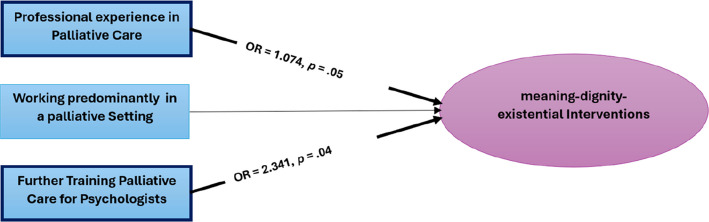
Binary logistic regression model for predicting the utilisation of meaning-dignity-existential interventions. The binomial logistic regression model was statistically significant, χ^2^(3) = 10.391, *p* =.02, with high goodness of fit (Hosmer–Lemeshow test, χ^2^(8) = 2.883, *p* >.05), but with very low variance explained by Nagelkerke’s *R*.^*2*^ =.079. Professional experience in the palliative setting had a positive influence on the use of meaning-dignity-existential interventions, with an odds of 1.074 (95% CI [1.002, 1.152]); further training in palliative care for psychologists had a positive influence on the use of meaning-dignity-existential interventions, with an odds of 2.341 (95% CI [1.025, 5.349]), *N* = *203*

As an intermediate step a theory-driven intuitive scale (see Appendix B—Table) was calculated from the mean values of the items PI33, PI34, PI35, PI36, PI37, PI38, PI39, PI40 and PI45 (*M* = 2.65, *MIC* = 0.325, *Cronbach’s α* = 0.810). The sample included *n* = 41 participants who fell into the category ‘meaning-dignity-existential’ high and *n* = 169 into the category ‘meaning-dignity-existential’ low. The assumptions of independent observations, no perfect multicollinearity and approximately linear relationship between the continuous predictor (PE) and the logit of the outcome were met. The statistically significant model [χ2(3) = 10.391, *p* = 0.02] demonstrated a high level of goodness of fit [Hosmer–Lemeshow test: χ2(8) = 2.883, *p* > 0.05], but a low level of variance explanation (Nagelkerke’s *R*^*2*^ = 0.079) and 79.8% correctly classified cases (without predictors 80.3%) (see Fig. 3 and Appendix D—Table). The classification plot with a cut-off at 0.50 showed that cases were incorrectly predicted, particularly in areas with high values on the ‘meaning-dignity-existential’ scale (with a sensitivity of 2.5% and a specificity of 98.8%). Model estimates suggested that, for every additional year of PE, the odds for using ‘meaning-dignity-existential’ interventions are approximately 1.074 higher (CI 95% [1.002, 1.152]). It became also apparent, that the odds for using ‘meaning-dignity-existential’ interventions were for participants with PCP approximately 2.341 higher compared with those without PCP (CI 95% [1.025, 5.349]). Working in a PS showed no significant influence in this sample.

### Insight into the reasons why interventions were rather rarely, rarely or never used

The most common reason (see Appendix E – Table and Appendix H—Figure) given by participants for rather rarely, rarely or never using an intervention was ‘not competent enough’ (*n* = 1299). This applied particularly, for example, to ‘catathymic-imaginative psychotherapy’ (*n* = 95), ‘trauma therapeutic interventions’ (*n* = 93), ‘hypnotherapeutic interventions’ (*n* = 84) or ‘manualised meaning-centred interventions CALM/SMiLE/Outlook’ (*n* = 55). The second most common reason was ‘I don’t know’ (*n* = 1022). This reason was often given for example regarding ‘systemic palliative psychotherapy’ (*n* = 121), ‘manualised meaning-centred interventions according to William Breitbart’ (*n* = 120), ‘manualised existential psychotherapy’ (*n* = 82) or ‘elements of life review approaches’ (*n* = 65). Other reasons such as ‘Not enough resources’ (*n* = 321) most frequently for ‘manualised dignity-centred therapy’ (*n* = 61), ‘Palliative care too short’ (*n* = 278) most frequently for ‘manualised dignity-centred therapy’ (*n* = 26), ‘Patients/relatives rarely suitable for it’ (*n* = 272) most frequently for ‘guideline-based psychological diagnostics/screening’ (*n* = 41) and ‘Patient usually no longer able to’ (*n* = 193) most frequently for ‘interventions from systemic therapy and counselling’ (*n* = 24) were less common. Other reasons from free text fields (*n* = 519) resulted in six categories with similar content (‘not my therapeutic attitude/orientation’, ‘not my job/someone else does it’, ‘not familiar with’, ‘not necessary/not required’, ‘not suitable in this setting’, ‘lack of resources/inappropriate structures’) and are summarized in Appendix F—Table.

## Discussion

### Key findings

To our knowledge, this is the first Germany-wide survey of psychologists and psycho-oncologists investigating the use and evaluation of psychological interventions in palliative care. Even if these results cannot be generalised and applied to other countries, they nevertheless provide a useful overview of how frequently different psychological interventions are used and why certain interventions are used less often (see Table 4). The most frequently used interventions include e.g. explorative diagnostics without guidelines, resource collection, psychological counselling, exploration of previous coping strategies, crisis intervention, or psychoeducation. Least frequently used were e.g. manualised existential psychotherapy, manualised meaning-centred interventions according to Breitbart, manualised dignity-centred therapy, manualised life review approaches or elements of meaning-centred interventions. Methods such as promoting successful communication within the MPCT, MBSR interventions, behavioural therapy elements or systemic therapy interventions and counselling were in the middle range and were used occasionally, rather rarely or rarely.

In summary, our findings indicate that interventions from a *specific palliative psychological approach* were used less frequently compared with non-specific interventions than expected and recommended. The most common reason was a lack of familiarity with such interventions, a lack of confidence in their ability to perform them competently or insufficient resources. Compared to the meaning-, dignity-centred and existential interventions, those from the (hypno)systemic-integrative approach appear to be more common yet were still used less frequently than expected. The most common reasons given for this were that participants either did not feel competent enough in this area, or that they had the impression that patients were no longer capable of participating or that palliative care was too short. Having longer professional experience in palliative care, as well as further training in palliative care for psychologists, increased the likelihood of meaning-dignity-existential interventions being used. The use of (hypno)systemic-integrative interventions only increased with longer professional experience.

### Discussion of the descriptive results

The most frequently used interventions can best be classified as general, low-threshold and non-specific. This type of interventions was also predominantly identified by psychologists in a German palliative hospital support team in 2024 [30]. This seems insofar plausible as this is an approach that is needs-oriented, resource-oriented, situation-adapted, and which is also recommended for this setting [32, 35]. A survey from Spain [110] reported also the use of interventions that were primarily non-specific and rather general in nature, like coping/adapting with the illness and the situation, integration of spiritual aspects or supporting communication between patients and their relatives. Specific meaning- and dignity-centred interventions or interventions from systemic counselling and therapy were probably not mentioned or named at the time (2008) due to a lack of awareness. In the UK, palliative psychologists mentioned *specific palliative psychological approaches* only in passing and played a subordinate role or were not reported at all [21, 24]. Another study described that interventions particularly suited to palliative care were unknown to the majority of the participants [111]. Among other things, the authors of the aforementioned studies conclude that greater consideration should be given to the specific needs in palliative care settings and that targeted interventions and approaches designed for this purpose should be integrated and evaluated [21, 24, 110, 111]. Given these long-standing conclusions and recommendations, it is noteworthy that meaning- and dignity-centred interventions were still little known and used relatively rarely in our study. The unsystematic research in this area and calls for specifying exactly what kind of psychological interventions were involved, who was conducting them or how symptoms were identified has been criticised [23]. Madl et al.’s proposal for psycho-oncology to develop a taxonomy of intervention techniques could be very useful for this purpose [112]. Such a comprehensive and structured taxonomy of palliative psychological intervention methods could lead to a common standard, facilitating care and efficacy research and increase the construct and content validity of a future survey. At the same time, it will probably remain challenging to operationalise the work of palliative psychologists in the same way as in other psychological or psychotherapeutic settings with standardised or manualised procedures. For this reason, several authors recommend tailoring and modifying the range of interventions to the needs and resources of those affected [40, 58, 71, 82, 113, 114]. Strada has developed a comprehensive framework for palliative psychology and offers useful guidance on this topic [32]. Such a framework concept based on evidence and expert knowledge that provides practitioners with practical guidelines would be an important and desirable step towards ensuring appropriate and comparable psychological support at the end of life. Implementing such recommendations and framework conditions could help make the training of psychologists and psycho-oncologists more targeted and clarify the remit of palliative care institutions with regard to palliative psychological support. Furthermore, it remains unclear to what extent the utilisation behaviour of those affected influences the use and evaluation of the interventions, as this could not be considered in this study. This potentially depends on patients’ perceived distress and need, attitudes toward psychological care, personality traits and sociodemographic factors, as well as on clinicians’ recommendations, information provision, and structural accessibility of services [115–117].

In light of the substantial evidence and recommendations for the targeted use of meaning- and dignity-centred interventions with seriously ill and dying patients [32, 86, 118, 119], the findings of this study prompt the question as to why such interventions remain underutilised and relatively unknown. Wishes for hasten death are a common symptom of psychological and existential distress experienced at the end of life [8]. They are often caused and perpetuated by feelings of loss of dignity, meaning, hope or autonomy, or by feeling like a burden to others [5, 120, 121]. Meaning- and dignity-centred interventions can alleviate not only existential distress but also reduce wishes for hasten death [47, 53, 122–125]. In view of the rising number of assisted dying [126, 127], such psychological interventions should be promoted and employed much more strongly as an effective contribution to successful suicide prevention in the palliative care context.

### Discussion of the inferential results

One possible explanation for the underrepresentation of interventions from a *specific palliative psychological approach* is a lack of relevant professional experience in palliative care and further training in palliative care for psychologists. This can potentially be interpreted as confirmation that a targeted knowledge transfer on dealing with serious physical illnesses, dying, death and grief continue to play a subordinate role within the field of psychology and psychotherapy [40]. It may also reflect a predominantly deficit-oriented focus on coping with declining physical and mental functioning. Existential concerns, particularly those related to dignity, meaning-making, and living in the face of death, remain comparatively neglected. Our sample showed that psychologists/psycho-oncologists with increased professional experience in palliative care and further training in palliative care for psychologists were more likely to use interventions centred on meaning, dignity and existential issues. For the more frequent use of (hypno)systemic-integrative methods, this only applied to those with longer professional experience. The finding that specialist training and professional experience seem to be essential for the perception and shaping of one’s own role and thus also influence the choice and use of interventions confirms previous research [25, 40, 114, 128]. Overall, these findings provide valuable information for potential palliative psychologists, can contribute to raising awareness among relevant stakeholders as well as educational institutions and serve as a valuable resource for future research projects. Uniform documentation of psychological consultations could serve as a quality indicator, unfold a positive effect on users themselves, in terms of self-reflection and learning from one another, but also on other professional groups [129]. This could help to make the work of palliative psychologists more transparent.

## Strengths and limitations

When interpreting the present results, the limitations of this study must also be considered. Although more than 200 [37] psychologists in Germany have completed further training in palliative care for psychologists, 134 [130] are currently certified as specialist psychologists for palliative care and 142 [131] registered with the German Society for Palliative Medicine psychology section, these figures are not reflected in the present study. As it was not possible to identify and examine the entire population of palliative psychologists in Germany, the extent to which the results are biased by participant self-selection remains unclear. It also remains unclear whether certain groups of psychologists/psycho-oncologists were not included because of the recruitment method. Accordingly, it is conceivable that those who could not be reached might have different levels of professional experience and qualification profiles and therefore different intervention preferences. We have addressed this obstacle by conducting an extensive search and contacting all identifiable institutions and potential psychologists/psycho-oncologists. Although the focus group discussion enabled a significant reduction of psychological interventions in the questionnaire (Appendix G – Figure), completion still took quite a long time which could have led to premature termination. Future studies of this kind should favour a more economical survey method. At the same time, this limitation and selection may also have led to important interventions not being considered. Even though our questions were formulated in such a way that the interventions surveyed could be used for both patients and relatives, it was left up to the participants to decide which of these two groups they referred to in each case. Future studies could benefit from focusing on one of the two target groups, as the repertoire of interventions is likely to differ in some respects. The interpretation of the results is further limited by the operationalisation of the variables of interest and the retrospective decision to use theory-driven intuitive scale construction for the inferential statistical analyses. In addition, valuable information may have been lost due to the scale ‘meaning-dignity-existential’ being dichotomised [132]. However, this approach appeared to be unavoidable, given the distribution of the data and a lack of suitable taxonomy. Methodological and statistical decisions also limit the generalisability of our results and interpretations. Given the heterogeneity and diversity of the qualifications and further training of the study participants other predictors, such as the existence of a licenced training programme, different therapy schools or other types of further training could have been tested (e.g. using multiple hierarchical regression). Predictor selection was theory-driven, based on the assumption that in the German context, specific training in palliative care may be more relevant for palliative psychological practice than other forms of professional training. Other qualifications (e.g. psychotherapy licensure) were analysed only exploratively and did not appear to influence intervention choice. Future research should examine these associations in greater detail. Despite its shortcomings, this survey has succeeded in offering valuable insights into palliative psychological work and thus has successfully closed this research gap at a national level.

## Conclusion

Low-threshold, needs- and resource-oriented psychological interventions are certainly effective and widely used to help palliative patients and their informal carers to better cope with physical, psychological, social, and spiritual distress. However, interventions specifically designed to address and alleviate existential distress, by strengthening the sense of dignity, fostering meaning, or providing comfort in inconsolable situations, seem to be comparatively less well known and thus less frequently applied. Existential suffering at the end of life can be caused and perpetuated by mental health conditions, but it is also often a reaction to the loss of dignity, meaning, hope, autonomy, control, or the inability to fulfil important roles. To respond to this type of psychospiritual suffering in a helpful and appropriate manner, psychologists and psycho-oncologists require a repertoire of interventions that enable those affected to (re)view themselves and their lives with dignity, meaning and coherence. Approaches such as life review, meaning- and dignity-centred interventions, logotherapy, existential analysis and systemic and hypnotherapeutic methods are suitable for alleviating precisely these aspects of suffering. More frequent use of these interventions would benefit not only patients and their relatives, but also practitioners. By focusing on aspects that broaden our understanding of the value and meaning of lived and remaining life, psychological care would act in the spirit of Cicely Saunders: ‘You matter because you are you, and you matter to the last moment of your life’ [133]. It would contribute to a core value at the heart of palliative care and holistic medicine.

## Supplementary Information

Supplementary Material 1.

## Data Availability

All data from this article are available as an appendix or on request.

## Abbreviations

MPCT: Multiprofessional palliative care teams
MBSR: Mindfulness-based stress reduction
CBT: Cognitive behavioural therapy
ACT: Acceptance and commitment therapy
PI: Psychological intervention
PIs: Psychological interventions
PCU: Palliative care units within hospitals
HST: Palliative care support teams within hospitals
SPHC: Specialist palliative home care
IH: Inpatient hospice
POS: Psychological/psycho-oncological services or liaison services
PE: Professional experience (years working as a psychologist/psycho-oncologist in a palliative care setting)
PCP: Further training in palliative care for psychologists
PS: Predominantly palliative setting
