# Supplementary material for: Use and evaluation of psychological interventions in specialist palliative care settings: results of a national online survey with psychologists and psycho-oncologists
Source: BMC Palliat Care. 2026 Mar 6;25:88. doi: 10.1186/s12904-026-02041-z (PMC13064260; doi:10.1186/s12904-026-02041-z)
Supplement: Supplementary file 1 — Supplementary Material 1. [file 12904_2026_2041_MOESM1_ESM.docx]

**Appendix information – Tables and Figures**

**For manuscript:** **Use and Evaluation of Psychological Interventions in Specialist Palliative Care Settings: Results of a National Online Survey with Psychologists and Psycho-oncologists.**

**Authors:** Ricarda Scheiner^1^, Isabel Burner-Fritsch^1^, Martin Fegg^1,2^, Berend Feddersen^1^, Claudia Bausewein^1^

^1^Department of Palliative Medicine, LMU University Hospital, Munich, Germany

^2^ Psychotherapy Clinic, Prof. Dr. Fegg and Colleagues, Sonnenstr. 10, 80331 Munich

*Corresponding address*

**Ricarda Scheiner**

Psychologist MSc, Specialist Psychologist for Palliative Care (BDP-DGP), Systemic Therapist for Individuals, Couples and Families (DGSF)

**Department of Palliative Medicine LMU University Hospital, Munich Germany**

Marchioninistr. 15, 81377 München

Tel.: +49 (0)89 4400 77926

Mobil: +49 (0) 1525 4848957

Fax: +49 (0)89 4400 74939

E-Mail: [ricarda.scheiner@med.uni-muenchen.de](mailto:ricarda.scheiner@med.uni-muenchen.de)

 www.palliativmedizin-muenchen.de

Appendix A - Table

*Intercorrelations (pairwise case exclusion): according to Pearson for metric variables (Age, PE) and for point-biserial correlation for dichotomous variables with ordinal variables (PS, PCP, PI01 to PI45), contingency measure Phi for dichotomous variables (PS, PCP); according to Spearman for correlations between metric (Age, PE) and ordinal variables (PI01 to PI45) and between ordinal variables (PI01 to PI45); PE =professional experience, PS = Palliative setting, PCP = Palliative Care for Psychologists. (N = 210)*

|  | **Age** | **PE** | **PS** | **PCP** | **PI01** | **PI02** | **PI03** | **PI04** | **PI05** | **PI06** | **PI07** | **PI08** | **PI09** | **PI10** | **PI11** | **PI12** | **PI13** | **PI14** | **PI15** | **PI16** | **PI17** | **PI18** | **PI19** | **PI20** | **PI21** | **PI22** | **PI23** | **PI24** | **PI25** | **PI26** | **PI27** | **PI28** | **PI29** | **PI30** | **PI31** | **PI32** | **PI33** | **PI34** | **PI35** | **PI36** | **PI37** | **PI38** | **PI39** | **PI40** | **PI41** | **PI42** | **PI43** | **PI44** | **PI45** |
| --- | --- | --- | --- | --- | --- | --- | --- | --- | --- | --- | --- | --- | --- | --- | --- | --- | --- | --- | --- | --- | --- | --- | --- | --- | --- | --- | --- | --- | --- | --- | --- | --- | --- | --- | --- | --- | --- | --- | --- | --- | --- | --- | --- | --- | --- | --- | --- | --- | --- |
| **Age** | - |  |  |  |  |  |  |  |  |  |  |  |  |  |  |  |  |  |  |  |  |  |  |  |  |  |  |  |  |  |  |  |  |  |  |  |  |  |  |  |  |  |  |  |  |  |  |  |  |
| **PE** | **.51^**^** | - |  |  |  |  |  |  |  |  |  |  |  |  |  |  |  |  |  |  |  |  |  |  |  |  |  |  |  |  |  |  |  |  |  |  |  |  |  |  |  |  |  |  |  |  |  |  |  |
| **PS** | .06 | **-.17^*^** | - |  |  |  |  |  |  |  |  |  |  |  |  |  |  |  |  |  |  |  |  |  |  |  |  |  |  |  |  |  |  |  |  |  |  |  |  |  |  |  |  |  |  |  |  |  |  |
| **PCP** | .03 | .13 | **.25**** | - |  |  |  |  |  |  |  |  |  |  |  |  |  |  |  |  |  |  |  |  |  |  |  |  |  |  |  |  |  |  |  |  |  |  |  |  |  |  |  |  |  |  |  |  |  |
| **PI01** | **- .25**** | -.10 | **-.24^**^** | -.04 | - |  |  |  |  |  |  |  |  |  |  |  |  |  |  |  |  |  |  |  |  |  |  |  |  |  |  |  |  |  |  |  |  |  |  |  |  |  |  |  |  |  |  |  |  |
| **PI02** | -.03 | .07 | -.01 | -.04 | **.16^*^** | - |  |  |  |  |  |  |  |  |  |  |  |  |  |  |  |  |  |  |  |  |  |  |  |  |  |  |  |  |  |  |  |  |  |  |  |  |  |  |  |  |  |  |  |
| **PI03** | .05 | -.01 | **.18^**^** | .11 | .03 | **.22^**^** | - |  |  |  |  |  |  |  |  |  |  |  |  |  |  |  |  |  |  |  |  |  |  |  |  |  |  |  |  |  |  |  |  |  |  |  |  |  |  |  |  |  |  |
| **PI04** | **.14^*^** | .04 | .01 | .01 | .09 | .13 | **.21^**^** | - |  |  |  |  |  |  |  |  |  |  |  |  |  |  |  |  |  |  |  |  |  |  |  |  |  |  |  |  |  |  |  |  |  |  |  |  |  |  |  |  |  |
| **PI05** | **.37^**^** | **.24^**^** | .01 | .07 | -.03 | **.26^**^** | .10 | **.38^**^** | - |  |  |  |  |  |  |  |  |  |  |  |  |  |  |  |  |  |  |  |  |  |  |  |  |  |  |  |  |  |  |  |  |  |  |  |  |  |  |  |  |
| **PI06** | .07 | **.19^**^** | -.11 | -.02 | .00 | .13 | .01 | **.28^**^** | **.29^**^** | - |  |  |  |  |  |  |  |  |  |  |  |  |  |  |  |  |  |  |  |  |  |  |  |  |  |  |  |  |  |  |  |  |  |  |  |  |  |  |  |
| **PI07** | -.07 | .06 | -.03 | .12 | .14^*^ | **.21^**^** | .00 | **.21^**^** | .11 | **.45^**^** | - |  |  |  |  |  |  |  |  |  |  |  |  |  |  |  |  |  |  |  |  |  |  |  |  |  |  |  |  |  |  |  |  |  |  |  |  |  |  |
| **PI08** | .07 | .11 | .02 | .05 | -.01 | .10 | .00 | .10 | .09 | **.20^**^** | .13 | - |  |  |  |  |  |  |  |  |  |  |  |  |  |  |  |  |  |  |  |  |  |  |  |  |  |  |  |  |  |  |  |  |  |  |  |  |  |
| **PI09** | **.18^**^** | .16^*^ | .08 | -.03 | .03 | .08 | -.05 | .07 | **.29^**^** | .13 | .05 | **.17^*^** | - |  |  |  |  |  |  |  |  |  |  |  |  |  |  |  |  |  |  |  |  |  |  |  |  |  |  |  |  |  |  |  |  |  |  |  |  |
| **PI10** | .02 | .07 | .01 | .04 | **.14^*^** | **.21^**^** | .02 | .12 | **.15^*^** | **.22^**^** | **.16^*^** | .10 | .08 | - |  |  |  |  |  |  |  |  |  |  |  |  |  |  |  |  |  |  |  |  |  |  |  |  |  |  |  |  |  |  |  |  |  |  |  |
| **PI11** | **.19^**^** | **.16^*^** | .10 | .05 | -.08 | .11 | .02 | .09 | **.18^*^** | **.17^*^** | .11 | **.18^*^** | **.16^*^** | **.31^**^** | - |  |  |  |  |  |  |  |  |  |  |  |  |  |  |  |  |  |  |  |  |  |  |  |  |  |  |  |  |  |  |  |  |  |  |
| **PI12** | .11 | .12 | .12 | .10 | -.12 | .10 | **.19^**^** | .17^*^ | **.20^**^** | .09 | -.05 | **.21^**^** | .17^*^ | **.27^**^** | **.45^**^** | - |  |  |  |  |  |  |  |  |  |  |  |  |  |  |  |  |  |  |  |  |  |  |  |  |  |  |  |  |  |  |  |  |  |
| **PI13** | **.27^**^** | **.24^**^** | .10 | -.02 | -.02 | .02 | .09 | .07 | **.26^**^** | .06 | -.06 | **.20^**^** | **.35^**^** | .09 | **.33^**^** | **.44^**^** | - |  |  |  |  |  |  |  |  |  |  |  |  |  |  |  |  |  |  |  |  |  |  |  |  |  |  |  |  |  |  |  |  |
| **PI14** | **-.20^**^** | -.13 | -.10 | -.03 | **.19^**^** | **.19^**^** | .06 | **.19^**^** | .08 | **.20^**^** | **.16^*^** | .06 | .12 | **.16^*^** | **.21^**^** | **.16^*^** | -.06 | - |  |  |  |  |  |  |  |  |  |  |  |  |  |  |  |  |  |  |  |  |  |  |  |  |  |  |  |  |  |  |  |
| **PI15** | **-.24^**^** | **-.16^*^** | -.04 | .06 | **.20^**^** | **.16^*^** | .00 | .01 | -.12 | .10 | **.22^**^** | .02 | -.06 | **.17^*^** | .05 | .05 | -.02 | **.28^**^** | - |  |  |  |  |  |  |  |  |  |  |  |  |  |  |  |  |  |  |  |  |  |  |  |  |  |  |  |  |  |  |
| **PI16** | .01 | .04 | .01 | -.02 | -.04 | .12 | .10 | **.22^**^** | .10 | .11 | **.22^**^** | **.17^*^** | .03 | .08 | **.15^*^** | .04 | -.04 | **.27^**^** | **.19^**^** | - |  |  |  |  |  |  |  |  |  |  |  |  |  |  |  |  |  |  |  |  |  |  |  |  |  |  |  |  |  |
| **PI17** | **.22^**^** | **.24^**^** | .08 | .13 | -.01 | -.03 | .09 | **.19^**^** | **.24^**^** | **.22^**^** | .11 | **.18^**^** | **.34^**^** | .10 | **.33^**^** | **.14^*^** | **.25^**^** | .07 | -.03 | **.27^**^** | - |  |  |  |  |  |  |  |  |  |  |  |  |  |  |  |  |  |  |  |  |  |  |  |  |  |  |  |  |
| **PI18** | **.14^*^** | .12 | .07 | .05 | -.06 | .09 | .03 | **.17^*^** | **.15^*^** | **.33^**^** | **.21^**^** | **.16^*^** | .13 | .04 | **.28^**^** | **.17^*^** | **.20^**^** | .13 | -.03 | **.21^**^** | **.33^**^** | - |  |  |  |  |  |  |  |  |  |  |  |  |  |  |  |  |  |  |  |  |  |  |  |  |  |  |  |
| **PI19** | **.23^**^** | **.22^**^** | -.04 | .16^*^ | -.04 | .02 | .03 | .08 | .12 | .12 | .13 | **.26^**^** | **.20^**^** | .02 | **.24^**^** | **.17^*^** | **.28^**^** | **.20^**^** | .09 | **.15^*^** | **.32^**^** | **.38^**^** | - |  |  |  |  |  |  |  |  |  |  |  |  |  |  |  |  |  |  |  |  |  |  |  |  |  |  |
| **PI20** | **.17^*^** | **.21^**^** | -.01 | .04 | -.10 | -.02 | -.05 | .09 | .12 | .12 | .08 | **.19^**^** | **.20^**^** | .01 | **.25^**^** | **.18^**^** | **.31^**^** | -.03 | -.10 | .11 | **.34^**^** | **.55^**^** | **.35^**^** | - |  |  |  |  |  |  |  |  |  |  |  |  |  |  |  |  |  |  |  |  |  |  |  |  |  |
| **PI21** | .04 | .09 | -.03 | .13 | .05 | .00 | .04 | -.02 | .13 | .05 | -.05 | .07 | **.20^**^** | .06 | .11 | .11 | **.36^**^** | -.06 | -.02 | -.02 | **.19^**^** | **.29^**^** | **.14^*^** | **.44^**^** | - |  |  |  |  |  |  |  |  |  |  |  |  |  |  |  |  |  |  |  |  |  |  |  |  |
| **PI22** | **.23^**^** | **.26^**^** | -.07 | .08 | -.13 | .03 | .08 | **.16^*^** | **.29^**^** | **.14^*^** | .11 | **.18^*^** | .13 | -.05 | .06 | .09 | **.17^*^** | .04 | -.12 | **.21^**^** | **.32^**^** | **.29^**^** | **.34^**^** | **.37^**^** | **.19^**^** | - |  |  |  |  |  |  |  |  |  |  |  |  |  |  |  |  |  |  |  |  |  |  |  |
| **PI23** | **.30^**^** | **.40^**^** | -.05 | **.15^*^** | **-.16^*^** | .00 | .06 | .08 | **.26^**^** | **.24^**^** | .10 | **.15^*^** | **.23^**^** | .02 | .14 | **.16^*^** | **.27^**^** | -.07 | -.13 | .11 | **.30^**^** | **.26^**^** | **.28^**^** | **.30^**^** | **.22^**^** | **.67^**^** | - |  |  |  |  |  |  |  |  |  |  |  |  |  |  |  |  |  |  |  |  |  |  |
| **PI24** | -.12 | -.12 | -.08 | .05 | **.23^**^** | **.14^*^** | -.00 | **.14^*^** | .13 | **.22^**^** | **.15^*^** | .12 | -.02 | .12 | **.18^**^** | .11 | .01 | **.35^**^** | .13 | **.20^**^** | .13 | **.25^**^** | .11 | .09 | -.07 | .11 | -.03 | - |  |  |  |  |  |  |  |  |  |  |  |  |  |  |  |  |  |  |  |  |  |
| **PI25** | **-.14^*^** | -.11 | -.07 | -.08 | **.20^**^** | .13 | -.08 | .13 | .12 | **.22^**^** | **.17^*^** | -.03 | -.02 | **.18^*^** | .13 | .01 | .01 | **.34^**^** | .10 | .09 | **.14^*^** | **.21^**^** | .07 | **.19^**^** | .05 | **.15^*^** | .03 | **.66^**^** | - |  |  |  |  |  |  |  |  |  |  |  |  |  |  |  |  |  |  |  |  |
| **PI26** | -.10 | -.10 | -.01 | .04 | .09 | -.02 | -.01 | .05 | .08 | **.20^**^** | **.23^**^** | .11 | .04 | .05 | **.22^**^** | .06 | .05 | **.34^**^** | **.23^**^** | **.27^**^** | **.22^**^** | **.23^**^** | **.24^**^** | **.14^*^** | -.03 | **.21^**^** | .09 | **.45^**^** | **.39^**^** | - |  |  |  |  |  |  |  |  |  |  |  |  |  |  |  |  |  |  |  |
| **PI27** | .11 | **.22^**^** | **-.21^**^** | .05 | **.14^*^** | .05 | .05 | .03 | .04 | .08 | **.15^*^** | .08 | .11 | .12 | .13 | .05 | **.16^*^** | .10 | .13 | .12 | **.18^*^** | **.17^*^** | **.21^**^** | **.25^**^** | **.20^**^** | **.25^**^** | **.22^**^** | .04 | .04 | **.18^**^** | - |  |  |  |  |  |  |  |  |  |  |  |  |  |  |  |  |  |  |
| **PI28** | .10 | **.15^*^** | **-.16^*^** | -.01 | .04 | -.05 | -.12 | -.07 | -.04 | .07 | .04 | **.17^*^** | **.22^**^** | -.02 | **.22^**^** | .07 | **.21^**^** | .11 | .06 | .09 | **.30^**^** | **.22^**^** | **.24^**^** | **.38^**^** | **.23^**^** | **.28^**^** | **.29^**^** | .10 | .12 | **.24^**^** | **.43^**^** | - |  |  |  |  |  |  |  |  |  |  |  |  |  |  |  |  |  |
| **PI29** | .07 | .06 | -.06 | .04 | **.14^*^** | .03 | -.05 | -.01 | .12 | .13 | .09 | .11 | **.28^**^** | .00 | **.18^*^** | .09 | **.34^**^** | .10 | **.14^*^** | .02 | **.41^**^** | **.22^**^** | **.32^**^** | **.25^**^** | **.24^**^** | **.20^**^** | **.19^**^** | .10 | **.15^*^** | **.24^**^** | **.29^**^** | **.29^**^** | - |  |  |  |  |  |  |  |  |  |  |  |  |  |  |  |  |
| **PI30** | .02 | .07 | -.06 | **.16^*^** | .02 | -.03 | -.03 | -.10 | -.03 | -.04 | .07 | **.25^**^** | **.17^*^** | -.06 | **.22^**^** | **.18^**^** | **.35^**^** | -.01 | -.01 | -.02 | **.23^**^** | **.19^**^** | **.27^**^** | **.38^**^** | **.37^**^** | **.22^**^** | **.23^**^** | .11 | .07 | .14 | **.16^*^** | **.29^**^** | **.40^**^** | - |  |  |  |  |  |  |  |  |  |  |  |  |  |  |  |
| **PI31** | -.07 | -.04 | -.09 | -.07 | **.22^**^** | **.16^*^** | .04 | .00 | .01 | **.23^**^** | **.31^**^** | **.18^**^** | .00 | **.19^**^** | **.14^*^** | .04 | .10 | **.28^**^** | **.17^*^** | **.19^**^** | .09 | **.24^**^** | **.29^**^** | **.19^**^** | .09 | **.25^**^** | **.17^*^** | **.31^**^** | **.34^**^** | **.39^**^** | **.26^**^** | **.23^**^** | **.31^**^** | **.22^**^** | - |  |  |  |  |  |  |  |  |  |  |  |  |  |  |
| **PI32** | -.03 | .06 | -.04 | .05 | **.28^**^** | .08 | .07 | -.02 | .02 | **.21^**^** | **.22^**^** | **.21^**^** | **.18^*^** | **.16^*^** | **.17^*^** | .05 | .11 | .11 | **.14^*^** | .11 | **.16^*^** | **.15^*^** | **.16^*^** | .09 | .14 | **.18^*^** | **.24^**^** | **.20^**^** | **.24^**^** | **.24^**^** | **.23^**^** | **.35^**^** | **.32^**^** | .07 | **.49^**^** | - |  |  |  |  |  |  |  |  |  |  |  |  |  |
| **PI33** | .02 | .04 | .11 | **.26^**^** | .07 | -.05 | .01 | -.07 | .04 | .05 | .09 | .12 | .08 | -.03 | **.22^**^** | .02 | **.15^*^** | .01 | .05 | -.02 | **.17^*^** | .07 | .10 | **.18^*^** | **.24^**^** | .12 | .13 | .05 | -.03 | **.21^**^** | .06 | **.23^**^** | **.17^*^** | **.29^**^** | **.17^*^** | **.19^**^** | - |  |  |  |  |  |  |  |  |  |  |  |  |
| **PI34** | .00 | .10 | .10 | **.22^**^** | .10 | **.15^*^** | .04 | -.04 | .05 | **.21^**^** | **.26^**^** | **.26^**^** | .08 | .14 | .29^**^ | .12 | **.17^*^** | .07 | .15^*^ | .12 | **.19^**^** | **.17^*^** | **.16^*^** | **.18^**^** | **.25^**^** | .07 | .08 | .07 | .01 | **.27^**^** | **.19^**^** | **.22^**^** | **.20^**^** | **.27^**^** | **.25^**^** | **.22^**^** | **.51^**^** | - |  |  |  |  |  |  |  |  |  |  |  |
| **PI35** | -.05 | .05 | -.10 | **.15^*^** | **.16^*^** | .03 | .02 | -.06 | -.03 | .05 | .09 | .13 | .11 | .13 | .12 | .12 | **.17^*^** | .00 | .01 | -.12 | .08 | .05 | .13 | **.18^**^** | **.33^**^** | .07 | .07 | .09 | .08 | **.21^**^** | **.27^**^** | **.26^**^** | **.25^**^** | **.41^**^** | .12 | .13 | **.31^**^** | **.35^**^** | - |  |  |  |  |  |  |  |  |  |  |
| **PI36** | **.20^**^** | **.21^**^** | -.12 | .14 | .14 | .02 | -.12 | -.09 | .11 | .07 | .09 | **.17^*^** | **.22^**^** | .03 | .12 | .09 | **.22^**^** | -.09 | .06 | -.05 | **.21^**^** | .12 | **.14^*^** | **.25^**^** | **.21^**^** | .10 | .13 | .04 | -.01 | .05 | **.34^**^** | **.24^**^** | **.30^**^** | **.27^**^** | .10 | **.20^**^** | **.37^**^** | **.38^**^** | **.51^**^** | - |  |  |  |  |  |  |  |  |  |
| **PI37** | **.17^*^** | **.32^**^** | -.12 | .11 | .12 | .13 | -.03 | -.02 | .22^**^ | .13 | .11 | **.24^**^** | **.22^**^** | **.22^**^** | **.22^**^** | **.14^*^** | **.15^*^** | -.02 | -.00 | .09 | **.25^**^** | .14 | .12 | **.22^**^** | **.14^*^** | **.25^**^** | **.20^**^** | .10 | .03 | .14 | **.31^**^** | **.19^**^** | **.28^**^** | **.22^**^** | **.20^**^** | **.26^**^** | **.26^**^** | **.45^**^** | **.42^**^** | **.66^**^** | - |  |  |  |  |  |  |  |  |
| **PI38** | .10 | .07 | -.00 | **.16^*^** | .01 | -.08 | .02 | -.01 | .06 | -.04 | -.01 | **.16^*^** | .09 | -.11 | .10 | **.15^*^** | **.22^**^** | -.06 | .02 | -.02 | .13 | .03 | .14 | **.18^**^** | **.22^**^** | **.20^**^** | **.17^*^** | .11 | .11 | .08 | **.21^**^** | **.29^**^** | **.27^**^** | **.37^**^** | .11 | **.17^*^** | **.35^**^** | **.26^**^** | **.39^**^** | **.50^**^** | **.34^**^** | - |  |  |  |  |  |  |  |
| **PI39** | .08 | .11 | .09 | .06 | .06 | .10 | .10 | .03 | .11 | .06 | .01 | **.22^**^** | .06 | .11 | **.18^*^** | .11 | **.18^*^** | .06 | .13 | .10 | **.20^**^** | **.15^*^** | .12 | **.14^*^** | **.14^*^** | **.19^**^** | **.15^*^** | **.16^*^** | **.17^*^** | **.20^**^** | **.17^*^** | **.21^**^** | **.22^**^** | **.27^**^** | **.19^**^** | **.17^*^** | .09 | **.35^**^** | **.24^**^** | **.29^**^** | **.44^**^** | **54^**^** | - |  |  |  |  |  |  |
| **PI40** | .02 | .05 | -.10 | .05 | .12 | -.12 | .02 | -.07 | -.05 | -.01 | .03 | .04 | .05 | -.01 | **.18^**^** | .11 | **.15^*^** | .02 | .08 | .09 | **.22^**^** | .06 | .09 | **.23^**^** | **.25^**^** | **.14^*^** | .09 | **.22^**^** | **.16^*^** | **.29^**^** | .13 | **.27^**^** | **.19^**^** | **.27^**^** | **.16^*^** | **.23^**^** | **.30^**^** | **.27^**^** | **.45^**^** | **.35^**^** | **.31^**^** | **.45^**^** | **.26^**^** | - |  |  |  |  |  |
| **PI41** | .02 | .03 | **.24^**^** | .02 | **-.14^*^** | .04 | .04 | .08 | **.14^*^** | .13 | .09 | **.19^**^** | .03 | **.23^**^** | **.40^**^** | **.25^**^** | **.31^**^** | .11 | .08 | .03 | **.19^**^** | **.19^**^** | .10 | **.20^**^** | **.14^*^** | .03 | .04 | .05 | **.14^*^** | **.14^*^** | .05 | **.16^*^** | **.16^*^** | **.14^*^** | **.20^**^** | **.19^**^** | .12 | **.31^**^** | .09 | .12 | **.20^**^** | .09 | **.18^**^** | .13 | - |  |  |  |  |
| **PI42** | .17^*^ | .13 | -.07 | .08 | .05 | -.11 | -.05 | -.00 | .02 | .06 | -.09 | -.04 | **.27^**^** | .02 | .06 | .08 | **.23^**^** | .10 | .01 | -.02 | .13 | -.01 | **.19^**^** | .07 | **.24^**^** | .08 | .11 | -.05 | .03 | .00 | **.26^**^** | **.18^*^** | **.28^**^** | .09 | .00 | **.17^*^** | **.20^**^** | .11 | **.26^**^** | **.21^**^** | **.14^*^** | **.25^**^** | .06 | **.28^**^** | .09 | - |  |  |  |
| **PI43** | **.18^**^** | .11 | .07 | .05 | .00 | -.12 | .04 | .03 | .08 | -.05 | -.00 | .14 | **.22^**^** | .02 | **.25^**^** | **.19^**^** | **.30^**^** | .03 | .02 | .02 | **.28^**^** | **.24^**^** | **.30^**^** | **.34^**^** | **.27^**^** | **.22^**^** | **.24^**^** | .01 | .02 | **.14^*^** | **.27^**^** | **.32^**^** | **.36^**^** | **.29^**^** | **.26^**^** | **.23^**^** | **.24^**^** | **.24^**^** | .08 | **.22^**^** | **.15^*^** | **.15^*^** | **.14^*^** | .10 | **.20^**^** | .13 | - |  |  |
| **PI44** | .06 | .11 | .05 | -.04 | -.05 | -.08 | .04 | .03 | **.17^*^** | .02 | -.02 | .07 | .12 | -.11 | .14 | -.06 | **.28^**^** | -.02 | -.05 | .07 | .13 | .10 | .05 | **.20^**^** | **.24^**^** | .12 | **.20^**^** | -.03 | -.02 | .03 | .09 | **.18^*^** | .11 | **.16^*^** | .08 | -.07 | **.20^**^** | **.17^*^** | .08 | **.15^*^** | .11 | .13 | .07 | .04 | **.17^*^** | .05 | **.17^*^** | - |  |
| **PI45** | **.15^*^** | **.16^*^** | .12 | .01 | -.11 | .08 | .01 | .01 | .13 | .11 | .12 | **.19^**^** | .05 | **.17^*^** | **.28^**^** | **.22^**^** | **.17^*^** | .14 | .11 | **.23^**^** | **.14^*^** | **.20^**^** | **.21^**^** | .12 | -.02 | **.19^**^** | .13 | .07 | .01 | **.19^**^** | .06 | **.14^*^** | .13 | **.19^**^** | **.26^**^** | .09 | .10 | **.19^**^** | .11 | .10 | .13 | .07 | **.20^**^** | .01 | **.25^**^** | -.05 | **.26^**^** | .12 | - |

*Notes.* **. The correlation is significant at the level of .01 (2-sided). *. The correlation is significant at the .05 level (2-sided). The correlation coefficients can be interpreted as effect size according to Cohen [^⁠1^](#_CTVL0018e326de9a64c46d78ec8bb4287ff8994): *r* = .10 to .20 ( small effect), *r* = .24 to .33 ( intermediate effect), *r* = .37 to .45 ( large effect), *r* ≥ .50 ( strong effect)

Appendix B - Table

*Mean values, standard deviations, standard errors of the mean values, median, skewness, kurtosis, range, Cronbach's α and inter-item correlation (MIC) for the items that were combined into scales (N = 210)*

|  | **Scale ‘(hypno)systemic-integrative’** (PI04,PI07,PI08,PI11,PI12,PI18,PI19,PI20,PI21,PI22,PI23) | **Scale ‘meaning-dignity-existential’** (PI33,PI34,PI35,PI36,PI37,PI38,PI39,PI40,PI45) |
| --- | --- | --- |
| number of items | 11 | 9 |
| *n** | 198 | 194 |
| *M* | 4.53 | 2.65 |
| *SD* | 0.81 | 1.11 |
| *SE* | .056 | .077 |
| *Median* | 4.5 | 2.3 |
| Skewness | -0.235 | 0.884 |
| Kurtosis | -0.143 | 0.073 |
| Range | 4.36 | 5.0 |
| *Cronbach’s α* | .761 | .810 |
| *MIC* | .220 | .325 |

*Notes.* *List-wise case exclusion for randomly missing values.

Appendix C - Table

*Results of the ordinal regression analysis with the predictor variables professional experience (metric), palliative setting (dichotomous) and further training in palliative care for psychologists (dichotomous) to predict the use of (hypno)systemic-integrative interventions (n = 203)*

| **Predictors** | ***β* (OR)** | ***p*** | **CI *β*** | ***SE*** | **Wald** | **Model fit** | ***Pseudo R^2^* (logit link)** |
| --- | --- | --- | --- | --- | --- | --- | --- |
| Professional Experience | **0.137 (1.147)** | < .001 | [0.084, 0.190] | 0.027 | 25.473 | χ²(3) = 30.481, *p* < .001 Pearson, χ²(3021) = 2693.376, *p* > .05  Deviation measure, χ²(3021) = 895.147, *p* > .05) | Cox & Snell = .139  Nagelkerke = .140  McFadden = .021 |
| Palliative Setting  less palliative setting | -0.308 (0.735) | .217 | [-0.796, 0.181] | 0.249 | 1.524 |  |  |
| Palliative Care for Psychologists  no further training | -0.490 (0.613) | .131 | [-1.126, 0.146] | 0.324 | 2.279 |  |  |

Notes.

Appendix D - Table

*Results of the binary logistic regression analysis with the predictor variables professional experience (metric), palliative setting (dichotomous) and further training in palliative care for psychologists (dichotomous) to predict the use of meaning-dignity-existential interventions (dichotomous), Method: inclusion, (n = 203)*

| **Predictors** | ***β*** | ***p*** | **OR** | **CI 95% OR** | ***SE*** | **Wald** | **Model fit** | ***Model summery and R^2^*** |
| --- | --- | --- | --- | --- | --- | --- | --- | --- |
| Professional Experience | 0.072 | .045 | **1.074** | [1.002, 1.152] | 0.36 | 4.016 | Omnibus test χ²(3) = 10.391, *p* = .02  Hosmer-Lemeshow test, χ²(8) = 2.883, *p* > .05 | -2  Log-  Likelihood = 191.097  Cox & Snell = .050  Nagelkerke = .079 |
| Palliative Setting  predominantly palliative setting | -0.342 | .361 | 0.710 | [0.341, 1.480] | 0.375 | 0.835 |  |  |
| Palliative Care for Psychologists  further training | 0.851 | .044 | **2.341** | [1.025, 5.349] | 0.422 | 4.072 |  |  |

*Notes.* Due to consistently non-normally distributed items (PI33,PI34,PI35,PI36,PI37,PI38,PI39,PI40,PI45), a dichotomisation of the dependent criterion variable scale ‘meaningful-dignified-existential’ was carried out for the inferential statistical analysis: Value tables 0= frequency low (never, almost never, rarely), 1= frequency high (very often, often, occasionally, rather rarely) -> cut-off at 3.5 out of 7; *n* = 41 fell into the category ‘meaningful dignity existential’ high and *n* = 169 into the category ‘meaningful dignity existential’ low

Appendix E - Table

*Response behaviour filter questions: if an intervention was rather rarely to never used, the reason for this was requested. If the appropriate reason was unavailable, 'Other reasons' could be selected, and participants could provide an individual reason in a free text field. (N = 210)*

|  |  | **Likert scale response options if an intervention was ‘rather rarely’ to ‘never’ used** | | | | | | |
| --- | --- | --- | --- | --- | --- | --- | --- | --- |
| **Item** | ***n*** | I don't know it (1) | not competent enough (2) | Patient/relatives rarely suitable for it (3) | Patient usually no longer able to (4) | Palliative care too short (5) | Not enough resources (6) | other reasons free text (7) |
| **PI01** ‘guideline-based psychological diagnostics/ screening’ | 98 | 2 | 3 | 41 | 8 | 13 | 5 | 26 |
| **PI02** ‘explorative diagnostics without guidelines’ | 7 | 2 | - | 2 | - | - | 1 | 2 |
| **PI03** ‘request for an order’ | 39 | 2 | 1 | - | - | - | 11 | 25 |
| **PI04** ‘order clarification’ | 3 | - | - | - | - | 1 | 1 | 1 |
| **PI05** ‘situation analysis/ behavioural analysis’ | 55 | 1 | 5 | 25 | 8 | 7 | 2 | 7 |
| **PI06** ‘exploration of previous coping strategies’ | 5 | - | 1 | 3 | - | - | 1 | - |
| **PI07** ‘resource collection’ | 2 | - | - | - | 1 | 1 | - | - |
| **PI08** ‘biography work’ | 32 | - | 5 | 3 | 4 | 12 | 6 | 2 |
| **PI09** ‘psychoanalytical exploration/ processing’ | 144 | 5 | 57 | 29 | 13 | 14 | 2 | 24 |
| **PI10** ‘psychological counselling’ | 1 | - | - | - | - | - | - | 1 |
| **PI11** ‘promote successful communication with patients and relatives’ | 19 | - | - | 3 | - | 7 | 2 | 7 |
| **PI12** ‘promote successful communication in the multiprofessional team’ | 55 | - | 4 | - | 1 | 3 | 14 | 33 |
| **PI13** ‘ethical counselling’ | 114 | 2 | 34 | 1 | - | 5 | 23 | 49 |
| **PI14** ‘psychoeducation’ | 21 | 1 | 3 | 8 | 4 | 1 | - | 4 |
| **PI15** ‘crisis intervention’ | 3 | - | - | - | - | 1 | - | 2 |
| **PI16** ‘client-centred conversation’ | 11 | - | 7 | - | - | - | - | 4 |
| **PI17** ‘elements from humanistic psychotherapy’ | 90 | 17 | 62 | 3 | - | 3 | - | 5 |
| **PI18** ‘systemic questions’ | 46 | 3 | 24 | 5 | 7 | 1 | 2 | 4 |
| **PI19** ‘impact techniques’ | 41 | 4 | 18 | 5 | 3 | 5 | 2 | 4 |
| **PI20** ‘interventions from systemic therapy and counselling’ | 130 | 4 | 49 | 14 | 24 | 20 | 8 | 11 |
| **PI21** ‘systemic palliative psychotherapy’ | 175 | 121 | 45 | - | 2 | 2 | 2 | 3 |
| **PI22** ‘hypnosystemic interventions’ | 130 | 36 | 69 | 6 | 9 | 5 | 2 | 3 |
| **PI23** ‘hypnotherapeutic interventions’ | 130 | 22 | 84 | 7 | 9 | 1 | 2 | 5 |
| **PI24** ‘cognitive elements from behavioural therapy’ | 44 | - | 16 | 10 | 5 | 5 | - | 8 |
| **PI25** ‘behavioural elements from behavioural therapy’ | 59 | - | 16 | 19 | 9 | 3 | 2 | 10 |
| **PI26** ‘interventions from acceptance and commitment therapy (ACT)’ | 32 | 4 | 21 | 2 | 1 | - | - | 4 |
| **PI27** ‘psycho-oncological interventions according to Diegelmann and Isermann’ | 125 | 61 | 41 | 9 | 3 | 4 | 5 | 2 |
| **PI28** ‘trauma therapeutic interventions’ | 167 | 7 | 93 | 17 | 19 | 17 | 6 | 8 |
| **PI29** ‘body psychotherapeutic interventions’ | 111 | 27 | 66 | 6 | 5 | 3 | 1 | 3 |
| **PI30** ‘interventions from embodiment research’ | 162 | 89 | 60 | 3 | 2 | 1 | 2 | 5 |
| **PI31** ‘interventions from MBSR’ | 50 | 4 | 17 | 6 | 8 | 6 | 4 | 5 |
| **PI32** ‘relaxation techniques’ | 40 | - | 5 | 4 | 8 | 7 | 7 | 9 |
| **PI33** ‘manualised dignity-centred therapy’ | 173 | 34 | 28 | 6 | 9 | 26 | 61 | 9 |
| **PI34** ‘elements from dignity-centred therapy’ | 117 | 33 | 23 | 3 | 5 | 23 | 25 | 5 |
| **PI35** ‘manualised meaning-centred interventions according to William Breitbart’ | 180 | 120 | 35 | 3 | 2 | 8 | 7 | 5 |
| **PI36** ‘manualised meaning-centred interventions (CALM, SMiLE, Outlook, etc.)’ | 154 | 74 | 55 | 1 | 1 | 10 | 7 | 6 |
| **PI37** ‘elements of meaning-centred interventions’ | 128 | 62 | 48 | - | 1 | 8 | 4 | 5 |
| **PI38** ‘manualised life review approaches’ | 155 | 67 | 30 | 4 | 3 | 20 | 16 | 15 |
| **PI39** ‘elements from life review approaches’ | 107 | 65 | 19 | 3 | 1 | 7 | 4 | 8 |
| **PI40** ‘manualised existential psychotherapy’ | 187 | 82 | 52 | 8 | 7 | 11 | 17 | 10 |
| **PI41** ‘grief counselling for relatives’ | 46 | 1 | 2 | - | - | 4 | 18 | 21 |
| **PI42** ‘catathymic-imaginative psychotherapy’ | 186 | 63 | 95 | 4 | 5 | 5 | 3 | 11 |
| **PI43** ‘other/creative methods/artistic therapies’ | 155 | 2 | 68 | 6 | 4 | 7 | 24 | 44 |
| **PI44** ‘animal-assisted therapy’ | 147 | 2 | 26 | 3 | 2 | 1 | 20 | 93 |
| **PI45** ‘integration of spiritual aspects into therapeutic activities’ | 28 | 3 | 12 | - | - | - | 2 | 11 |
| **Total** |  | 1022 | 1299 | 272 | 193 | 278 | 321 | 519 |

Appendix F - Table

*Content-oriented analysis of free text fields ‘other reason’ with deductive category formation. (n) participants ticked another reason and n participants actually stated another reason in the free text field. (N = 210)*

|  | *n* | not my therapeutic attitude/orientation | not my job/ someone else does it | not familiar with | not necessary/ not required | not suitable in this setting | lack of resources/ inappropriate structures |
| --- | --- | --- | --- | --- | --- | --- | --- |
| **PI01** ‘guideline-based psychological diagnostics/ screening’ | (26)  25 | 14 | 7 | 3 | 1 | 12 | 1 |
| ***Example of original Statement*** |  | *‘I prefer the personal diagnostic interview.’* | *‘The screenings are handed out by the nursing staff.’* | *‘As a doctor, I am not socialised to working with questionnaires.’* | *‘Not necessary to determine the patients' needs.’* | *‘In my experience, patients are often overwhelmed by questionnaires.’* | *‘Too few time resources in the hospice.’* |
| **PI02** ‘explorative diagnostics without guidelines’ | (2)  1 | - | - | - | - | 1 | - |
| ***Example of original Statement*** |  |  |  |  |  | *‘We have a different way of asking about needs; self-report questionnaires are not really suitable’* |  |
| **PI03** ‘request for an order’ | (25)  25 | 2 | 18 | 2 | - | 4 | 1 |
| ***Example of original Statement*** |  | *‘I only seek a psycho-oncological consultation if the patient requests one.’* | *‘The palliative care specialists and our doctors ask about wishes and needs.’* | *‘Not usual for us. Patients are admitted and automatically receive psycho-oncological care.’* |  | *‘Some patients would be overwhelmed by a specific order.’* | *‘I can't walk around the wards in a 600-bed hospital and pick out the palliative patients myself; I wait for my profession to request a consultation’.* |
| **PI04** ‘order clarification’ | (1)  1 | 1 | - | - | - | - | - |
| ***Example of original Statement*** |  | *‘I have difficulty with the term ‘order clarification’, I experience the work as primarily explorative, supportive and process-orientated due to the setting.’* |  |  |  |  |  |
| **PI05** ‘situation analysis/ behavioural analysis’ | (7)  6 | 5 | - | - | - | 2 | - |
| ***Example of original Statement*** |  | *‘I am a systemic psychotherapist and work in a more process-orientated way.’* |  |  |  | *‘I have (so far) usually found this intervention to be inappropriate in the palliative setting.’* |  |
| **PI08** ‘biography work’ | (2)  2 | - | - | - | - | 2 | - |
| ***Example of original Statement*** |  |  |  |  |  | *‘Because in most cases it has no added value for the patient.’* |  |
| **PI09** ‘psychoanalytical exploration/ processing’ | (24)  24 | 18 | - | - | - | 9 | 1 |
| ***Example of original Statement*** |  | *‘Does not correspond to my expertise and my self-image.’* |  |  |  | *‘I consider this to be helpful in the palliative situation in very few cases.’* | *‘I have too little time resources.’* |
| **PI10** ‘psychological counselling’ | (1)  1 | - | 1 | - | - | - | - |
| ***Example of original Statement*** |  |  | *‘Is mostly or exclusively - until now - carried out by the nursing staff, the hospice management and the doctors.’* |  |  |  |  |
| **PI11** ‘promote successful communication with patients and relatives’ | (7)  8* | - | 2 | - | 5 | - | 1 |
| ***Example of original Statement*** |  |  | *‘Family discussions are conducted by the medical, nursing and social staff.’* |  | *‘Is not required.’* |  | *‘Due to the exclusive consultation in the clinic, there is rarely an opportunity for this.’* |
| **PI12** ‘promote successful communication in the multiprofessional team’ | (33)  33 | - | 13 | - | 17 | 1 | 7 |
| ***Example of original Statement*** |  |  | *‘The senior physicians usually do this for us.’* |  | *‘The multiprofessional team is well trained and has regular external supervision.’* | *‘This usually feels inappropriate in my role/position in the team.’* | *‘Personnel resources, no fixed responsibility/affiliation to the team.’* |
| **PI13** ‘ethical counselling’ | (49)  49 | 2 | 33 | - | 14 | 1 | 1 |
| ***Example of original Statement*** |  | *‘I am not an ethics counsellor.’* | *‘We have an ethics committee at the hospital specifically for this purpose.’* |  | *‘No demand from patients so far.’* | *‘Individual medical practice’* | *‘I rarely have any contact with colleagues.’* |
| **PI14** ‘psychoeducation’ | (4)  2 | 2 | - | - | - | 1 | - |
| ***Example of original Statement*** |  | *‘I don't like the word 'psychoeducation' either.’* |  |  |  | *‘The palliative care setting is also not particularly conducive to 'lecturing' people.’* |  |
| **PI15** ‘crisis intervention’ | (2)  2 | - | - | - | 1 | - | 1 |
| ***Example of original Statement*** |  |  |  |  | *‘It hardly ever occurs.’* |  | *‘… we spend 8 hours a week on the palliative care ward, and I don't necessarily notice the crises, which are often dealt with by the nursing staff and doctors.’* |
| **PI16** ‘client-centred conversation’ | (4)  4 | 2 | - | 2 | - | - | - |
| ***Example of original Statement*** |  | *‘Licence in behavioural therapy → focus on these interventions.’* |  | *‘However, I am not familiar enough with Rogers to know his conditions etc. exactly.’* |  |  |  |
| **PI17** ‘elements from humanistic psychotherapy’ | (5)  5 | 5 | - | - | - | 1 | - |
| ***Example of original Statement*** |  | *‘There are concepts from depth psychology that are closer to me.’* |  |  |  | *‘Psycho-oncological work in acute hospitals often involves crisis intervention […] and should be distinguished from psychotherapy.’* |  |
| **PI18** ‘systemic questions’ | (4)  4 | 4 | - | - | - | - | - |
| ***Example of original Statement*** |  | *‘I don't feel very comfortable in the setting and with regard to my therapeutic identity.’* |  |  |  |  |  |
| **PI19** ‘impact techniques’ | (4)  4 | - | 1 | - | 1 | 2 | 1 |
| ***Example of original Statement*** |  |  | *‘Overlap with art/music therapy’* |  | *‘Just hasn't happened often yet.’* | *‘The approach often seems to be ‘too technical’ - just as behavioural therapy interventions as a whole seem to miss the mark.’* | *‘Lack of time.’* |
| **PI20** ‘interventions from systemic therapy and counselling’ | (11)  9 | 3 | 1 | - | 2 | 4 | 2 |
| ***Example of original Statement*** |  | *‘I work within depth psychology, supplemented by integrative psychotherapy.’* | *‘These techniques are used by other therapists in the team (music therapist, art therapist)’* |  | *‘The opportunity rarely arises, as other topics often take centre stage.’* | *‘Often goes too far in the situation of dying people. Other things are more urgent.’* | *‘The setting, e.g. the rooms (bedside conversation in a 4-bed room), does not allow for this. Unfortunately.’* |
| **PI21** ‘systemic palliative psychotherapy’ | (3)  3 | 2 | - | 1 | - | - | 1 |
| ***Example of original Statement*** |  | *‘I am not a psychotherapist.’* |  | *‘I have only recently read the book and have not yet been able to put the contents into practice for myself.’* |  |  | *‘Unfortunately, often due to time constraints’.* |
| **PI22** ‘hypnosystemic interventions’ | (3)  3 | 2 | - | - | - | 1 | - |
| ***Example of original Statement*** |  | *‘Because I don't have the appropriate training.’* |  |  |  | *‘Such therapeutic methods rarely correspond to the patient's/family member's described concerns.’* |  |
| **PI23** ‘hypnotherapeutic interventions’ | (5)  5 | 3 | 1 | -- | - | - | 1 |
| ***Example of original Statement*** |  | *‘I work with depth psychology.’* | *‘A trained registered nurse works with the patients here if required.’* |  |  |  | *‘Unfortunately, the general conditions (rooms, peace and quiet) are often not available.’* |
| **PI24** ‘cognitive elements from behavioural therapy’ | (8)  8 | 4 | - | - | - | 4 | - |
| ***Example of original Statement*** |  | *‘I am currently training to become a depth-psychological-psychoanalytical psychotherapist. The focus here is on engaging with the patient.’* |  |  |  | *‘My impression is that cognitive elements do not have a lasting effect in highly stressful situations.’* |  |
| **PI25** ‘behavioural elements from behavioural therapy’ | (10)  10 | 6 | - | - | - | 5 | - |
| ***Example of original Statement*** |  | *‘I am not a behavioural therapist.’* |  |  |  | *‘In the hospital setting in which I care for palliative patients, classically learning theory-based interventions are less helpful.’* |  |
| **PI26** ‘interventions from acceptance and commitment therapy (ACT)’ | (4)  4 | 2 | - | - | - | 2 | - |
| ***Example of original Statement*** |  | *‘As a depth psychologist, I do not use any other concepts, but elements of them that are also used in psycho-oncological training.’* |  |  |  | *‘I don't think it makes sense’* |  |
| **PI27** ‘psycho-oncological interventions according to Diegelmann and Isermann’ | (2)  2 | - | - | 2 | - | - | - |
| ***Example of original Statement*** |  |  |  | *‘I only have the relevant theoretical knowledge and no practical experience (yet) with the interventions mentioned.’* |  |  |  |
| **PI28** ‘trauma therapeutic interventions’ | (8)  6 | 2 | - | - | 2 | 2 | 2 |
| ***Example of original Statement*** |  | *‘I tend to work with ego states or with the inner team.’* |  |  | *‘This is not an issue in the context of my palliative work.’* | *‘Patient no longer able; accompaniment too short.’* | *‘And too little time.’* |
| **PI29** ‘body psychotherapeutic interventions’ | (3)  3 | - | 1 | 2 | - | - | - |
| ***Example of original Statement*** |  |  | *‘This is where physiotherapy comes in and takes up these structures.’* | *‘Simply not utilised to date, low experience of competence.’* |  |  |  |
| **PI30** ‘interventions from embodiment research’ | (5)  4 | 1 | - | 3 | - | - | - |
| ***Example of original Statement*** |  | *‘I can manage sufficiently with other methods.’* |  | *‘I don't know well enough.’* |  |  |  |
| **PI31** ‘interventions from MBSR’ | (5)  5 | 3 | - | - | - | 2 | - |
| ***Example of original Statement*** |  | *‘I prefer other interventions.’* |  |  |  | *‘The necessary tranquillity is usually not provided during hospitalisation.’* |  |
| **PI32** ‘relaxation techniques’ | (9)  8 | 3 | 1 | - | - | 4 | 1 |
| ***Example of original Statement*** |  | *‘Simply not my way of working.’* | *‘Does the physiotherapy.’* |  |  | *‘These techniques require either long periods of concentration or longer periods of rest or technical equipment.’* | *‘As I said, in 1-3 conversations...’* |
| **PI33** ‘manualised dignity-centred therapy’ | (9)  8 | 1 | 2 | 2 | - | 2 | 2 |
| ***Example of original Statement*** |  | *‘Manuals are stimulating, but I myself work with the elements that might suit the person.’* | *‘A trained dignity therapist will be called in if necessary.’* | *‘I know that this exists, but I have neither knowledge nor experience with the manual.’* |  | *‘Accompaniment too short.’* | *‘Too little time.’* |
| **PI34** ‘elements from dignity-centred therapy’ | (5)  5 | - | 1 | 1 | - | 2 | 3 |
| ***Example of original Statement*** |  |  | *‘Colleague’* | *‘I know that this exists, but I have neither knowledge nor experience with the manual.’* |  | *‘An inpatient setting with neighbouring patients in the same room is rather unsuitable for this.’* | *‘Too little time.’* |
| **PI35** ‘manualised meaning-centred interventions according to William Breitbart’ | (5)  3 | 2 | - | 1 | - | - | - |
| ***Example of original Statement*** |  | *‘I do not consider a manualised approach to be appropriate in this context.’* |  | *‘I know that this exists, but I have neither knowledge nor experience with the manual.’* |  |  |  |
| **PI36** ‘manualised meaning-centred interventions (CALM, SMiLE, Outlook, etc.)’ | (6)  5 | 3 | - | 1 | - | 1 | 1 |
| ***Example of original Statement*** |  | *‘Manualised procedure makes ‘being in relationship’ with the patient more difficult.’* |  | *‘I know that this exists, but I have neither knowledge nor experience with the manual.’* |  | *‘Too short accompaniment and other things take priority.’* | *‘Too little time.’* |
| **PI37** ‘elements of meaning-centred interventions’ | (5)  4 | 2 | - | 2 | - | - | - |
| ***Example of original Statement*** |  | *‘So far, I have had few points of contact with these interventions, which is why they are usually not cognitively present in the conversation.’* |  | *‘I suppose that I use individual elements, but I don't know the manuals.’* |  |  |  |
| **PI38** ‘manualised life review approaches’ | (15)  13 | 9 | 1 | 1 | - | 2 | 1 |
| ***Example of original Statement*** |  | *‘I have good experience with the questions of dignity-centred therapy.’* | *‘This is the task of my colleague.’* | *‘I don't know this like many of the manualised procedures, but I would also find a strong highly manualised approach to be inappropriate for contact with palliative care patients.’* |  | *‘Crisis intervention usually takes centre stage.’* | *‘Time.’* |
| **PI39** ‘elements from life review approaches’ | (8)  7 | 3 | 1 | 2 | - | 1 | 2 |
| ***Example of original Statement*** |  | *‘Questions on life review are partly included automatically, but not in manualised form.’* | *‘Colleague’* | *‘I have neither knowledge nor experience with the manual.’* |  | *‘Brevity of the encounter.’* | *‘Time’* |
| **PI40** ‘manualised existential psychotherapy’ | (10)  9 | 4 | - | 1 | 4 | - | 4 |
| ***Example of original Statement*** |  | *‘I am not a psychotherapist.’* |  | *‘I have neither knowledge nor experience with the manual.’* | *‘We do not hold group meetings.’* |  | *‘Group offers in the hospital are rather less possible.’* |
| **PI41** ‘grief counselling for relatives’ | (21)  21 | - | 8 | - | 13 | - | 7 |
| ***Example of original Statement*** |  |  | *‘Not my area of responsibility.’* |  | *‘Rarely occurs despite offers.’* |  | *‘Unfortunately, this is not provided for, there is no way to settle it.’* |
| **PI42** ‘catathymic-imaginative psychotherapy’ | (11)  10 | 7 | 1 | 1 | - | 1 | - |
| ***Example of original Statement*** |  | *‘It is not my psychotherapeutic orientation, I do not use this.’* | *‘We have an art therapist who works with it.’* | *‘I have neither knowledge nor experience with the manual.’* |  | *‘Medication also reduces the ability to concentrate, patients fall asleep quickly, method remains incomplete.’* |  |
| **PI43** ‘other/creative methods/artistic therapies’ | (44)  44 | - | 39 | 2 | 1 | 2 | 2 |
| ***Example of original Statement*** |  |  | *‘We have our own therapists for these programmes.’* | *‘I have no training in music therapy.’* | *‘No interest on the part of the seriously ill.’* | *‘Crisis intervention is rather common in everyday ward life.’* | *‘Not provided for in the structures.’* |
| **PI44** ‘animal-assisted therapy’ | (93)  93 | 3 | 9 | 5 | 14 | 4 | 69 |
| ***Example of original Statement*** |  | *‘Is absolutely not in my field of experience.’* | *‘Our ‘animal therapist’ comes on average 2 - 3 Saturdays a month.’* | *‘I don't have any further training in this area, nor do I have the necessary resources (animals).’* | *‘Not offered here.’* | *‘Instrumentalization of non-human animals is ethically unacceptable.’* | *‘Animals are not permitted on the ward/ in the clinic.’* |
| **PI45** ‘integration of spiritual aspects into therapeutic activities’ | (11)  11 | - | 11 | - | - | - | - |
| ***Example of original Statement*** |  |  | *‘Questions of faith and spiritual matters are covered by our pastoral care.’* |  |  |  |  |

*Notes.* For each intervention where ‘other reason’ was selected, the frequency of the reasons given is listed and an example text is quoted.


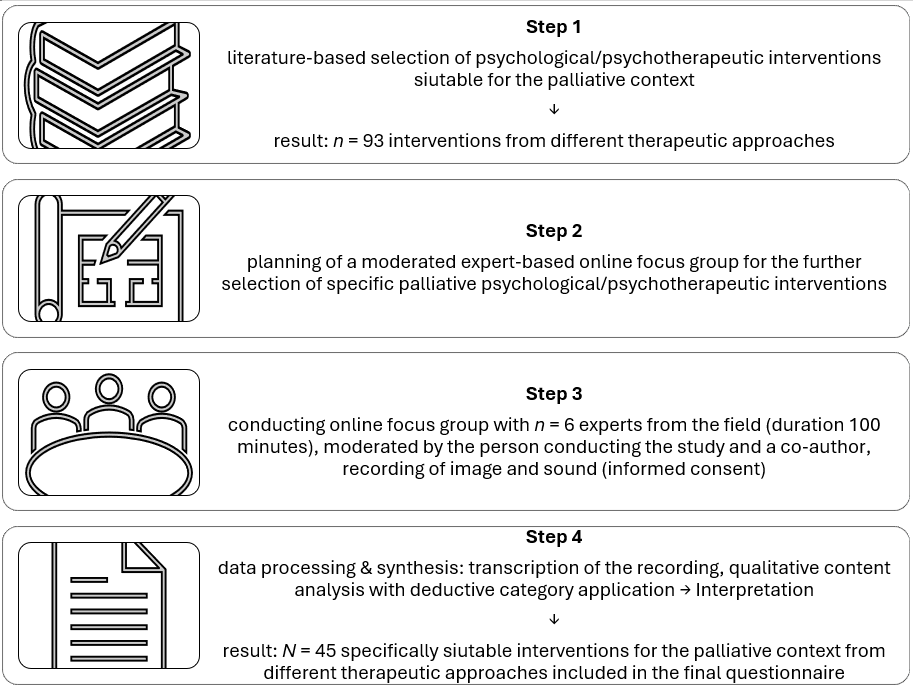


*Appendix G – Figure.* Selection process of psychological/psychotherapeutic interventions based on the use of a focus group discussion

*Appendix H – Figure.* Response behaviour filter questions: if an intervention was rather rarely (4) to never (1) used, the reason for this was requested. If the appropriate reason was unavailable, 'Other reasons' could be selected, and participants could provide an individual reason in a free text field. N = 210.

References

1. Cohen J. *Statistical power analysis for the behavioral sciences.* 2. ed. Hillsdale, NJ: Erlbaum, 1988.
